# Adaptive Impact Mitigation Based on Predictive Control with Equivalent Mass Identification

**DOI:** 10.3390/s23239471

**Published:** 2023-11-28

**Authors:** Cezary Graczykowski, Rami Faraj

**Affiliations:** Institute of Fundamental Technological Research Polish Academy of Sciences, Pawińskiego 5B, 02-106 Warsaw, Poland; rfaraj@ippt.pan.pl

**Keywords:** adaptive impact absorption, semi-active control, self-adaptive shock absorber, adaptive model predictive control, model identification adaptive control, model predictive control

## Abstract

The paper presents the concept of equivalent parameter predictive control (EPPC) elaborated for semi-active fluid-based (hydraulic and pneumatic) shock absorbers equipped with controllable valves and subjected to impact excitation. The undertaken problem concerns the absorption and dissipation of the impact energy with the requirement to minimize the generated reaction force and corresponding impacting object deceleration. The development of a control strategy for a challenging problem with unknown impacting object mass and unknown changes of external and disturbance forces is proposed and discussed in detail. The innovative solution utilizes the paradigm of model predictive control supplemented by the novel concept of equivalent system parameters identification. The EPPC is based on the online measurement of system response, the computation of the equivalent mass of the impacting object, and the repetitive solution of the optimal control problem with various prediction intervals and constraints imposed on valve opening. The presented method is proven to operate robustly for unknown excitations, including double-impact conditions, and it has similar efficiency to control methods developed previously for known impact parameters.

## 1. Introduction

Vibration damping and impact absorption are two important groups of problems in the field of dynamic excitation mitigation. Although the problem of impact absorption is important from a practical point of view and brings important research challenges, it is much less pronounced in the scientific literature. The requirement for efficient impact absorptions occurs in many mechanical systems present in various branches of contemporary engineering. In particular, in the case of the automotive industry, the problem of impact absorption is crucial, and novel technical solutions are being developed for car suspensions [1,2,3,4], internal and external car airbags [5], vehicles bumpers [6,7], and road barriers [8]. In the case of aviation and aeronautics, the typical application of shock absorbers pertains to aircraft landing gears [9,10,11], while less common but equally important are helicopter seat suspensions [12], airdrop systems [13,14], emergency landing airbags for drones [15], or suspensions of lunar-planetary landers [16]. In turn, within rescue applications, the effective protection of people falling from heights is provided by the use of impact detection systems [17] and safety airbags [18]. Moreover, impact mitigation devices are important for everyday applications, including the design of helmets [19], bicycle dampers and handlebars [20], and wearable airbags [21].

Similarly to the classification of vibration damping systems, the systems for impact absorption can be divided into three groups: passive, semi-active, and active. Novel passive and semi-passive impact-absorbing systems, based, e.g., on variable thread lead inerters [22], are still being developed due to their simple construction and the low energy required for efficient operation. On the other hand, passive systems are often being replaced by semi-active or active solutions. Semi-active systems of impact absorption can be divided into absorbers based on deformable materials (e.g., controllable elastic–plastic materials [23] or shape memory alloys [24]); devices based on impact and friction mechanisms, such as particle dampers [25,26,27]; biomimetic structures [28]; and fluid-based devices composed of cylinder and piston. The operation of the latter devices can be based either on the control of functional fluids’ properties (magnetorheological dampers [29,30,31,32] or electrorheological dampers [33]) or control of the standard fluid flow using fast operating valves with piezoelectric [34] and magnetostrictive actuators. The presented study is focused on the last group of cylinder piston devices and discusses an illustrative example of a pneumatic shock absorber.

The classical approach to impact mitigation problem, so-called adaptive impact absorption (AIA) [35], has been developed over the last two decades and requires a number of consecutive steps. First, the preliminary identification of impact excitation is conducted just before or at the beginning of the impact process. Then, the optimal impact mitigation scenario, which provides dissipation of the impact energy and the minimization of generated force, is determined. Finally, the mitigation of system response is executed using embedded control devices. Such an approach has been considered for various types of engineering structures [36], especially to adaptive pneumatic cylinders [37] and semi-active landing gears, which can utilize various types of feedback and various types of PID controllers [38,39,40].

Despite the fact that the control scenario can be realized using force or deceleration feedback, the AIA-based systems do not react properly to changes of loading conditions and do not compensate for possible inaccuracies of excitation identification. Therefore, the above classical AIA approach has been recently replaced by the innovative concept of self-adaptive systems [41]. The problem of impact absorption for the cylinder–piston system has been redefined as an optimal control problem aimed at the minimization of global discrepancy between the obtained and actually optimal system path, which is updated online during impact absorption process. Such an approach leads to the state-dependent path-tracking problem, which can be repeatedly solved at each control step in order to determine the actually optimal valve control. The simplified solution to the problem is hybrid prediction control (HPC) [42] based on combination of digital (on/off) and analogue (proportional) control, which has been tested numerically and validated experimentally using drop tests [43]. A more exact solution determined for the case of unknown excitation and disturbance force leads to identification-based predictive control (IPC) [44], which includes the identification of external forces, repeated at each control step, and sequential solution of the optimal control problem. Extension of the IPC method to identify leakages and mitigate their effects on the operation of a shock absorber equipped with a piezoelectric valve was proposed and investigated in [45].

This paper continues the development of self-adaptive control systems based on the state-dependent path-tracking problem. The considered challenging task is the elaboration of a control strategy for the case when neither all system parameters nor applied excitation are fully defined, i.e., the case of an unknown value of the impacting object mass and an unknown change of external and disturbance forces during the process. The above assumptions, resulting in a large number of unknown quantities, mean that the application of previously adopted identification methods and the straightforward derivation of the predictive model is no longer possible. Nevertheless, the proposed problem solution effectively utilizes a combination of the well-known concepts of model identification adaptive control and model predictive control. The control strategy is composed of two separate procedures repeated at each step of the process. The first one is the determination of the so-called equivalent mass of the impacting object, which takes into account the presence of external and disturbances forces. The second one is the application of the derived predictive model to solve the state-dependent path-tracking problem and to find the optimal valve opening for the assumed prediction interval, taking into account various control constraints. The proposed control method is especially effective in the case of changeable impacting object mass or additional inertial loading, so it can be successfully applied for the problem of double-impact excitation. Consequently, the method extends the range of impact mitigation problems considered previously in the literature and constitutes an important contribution to the field of adaptive impact absorption.

On the other hand, the above-described problem of control of the impact absorption process can be compared to the problem of motion control of various types of electric, hydraulic, and pneumatic actuators used, e.g., in manipulators and robots. Although both types of tasks have different objectives, their common feature is tracking the assumed kinematics, as well as the application of predictive and adaptive control techniques for obtaining a robust system response. The exemplary approaches include the usage of MPC in the hybrid actuator of the artificial muscle [46], application of the fuzzy sliding mode control for compliant rescue manipulator [47], and development of a hybrid actuation system for the upper limb exoskeleton [48]. Two noteworthy methods dedicated for motion control, i.e., robust fault-tolerant optimal predictive control with time-varying delay for a robot arm [49] and adaptive model predictive control of the hydraulic actuator [50], are compared against the control methods applied for impact absorption in Table 1.

The remainder of the paper is organized as follows. The second section introduces readers to the general problem of unknown impact mitigation, presents the analyzed double-chamber fluid-based absorber, and describes various versions of the state–space model of the considered system. The third section presents variational formulations of the state-dependent path-tracking problem for the case of an unknown mass and disturbance force, as well as its decomposition into a series of problems for receding control horizons. The fourth section introduces the concept of equivalent parameter predictive control, derives the applied predictive model, and proposes three different control implementations. The fifth section describes a dedicated sub-optimal analytical control strategy, which provides low cost of computations and convenient handling of valve operation constraints. Finally, the sixth section presents numerical examples, proving the efficiency of the EPPC for single- and double-impact excitation.

## 2. Considered Mechanical System and Its State–Space Model

### 2.1. Shock Absorber under Double-Impact Excitation

This study concerns more complex excitation conditions than considered so far in the research concerning adaptive impact absorption. The most-often-solved problems correspond to deceleration of a single object with initial velocity or under the influence of impulsive force, which are assumed to be a priori known or identified at the beginning of the impact process. In contrast, in this paper, the problem is extended to the case of entirely unknown impact excitation, where the mass of the impacting object, its initial velocity, and impulsive force are not preliminarily known by the impact mitigating system. The considered example of unknown impact excitation includes double-impact conditions, in which an impacting object decelerated by the shock absorber experiences an additional impact due to its collision with a second object of unknown parameters. Such a situation appears, e.g., during road accidents, where the first car hits an obstacle, and the second car impacts the first car. In the next sections, a control method ensuring efficient impact mitigation of the first as well as the second impact will be proposed and analyzed.

**Figure 1:**
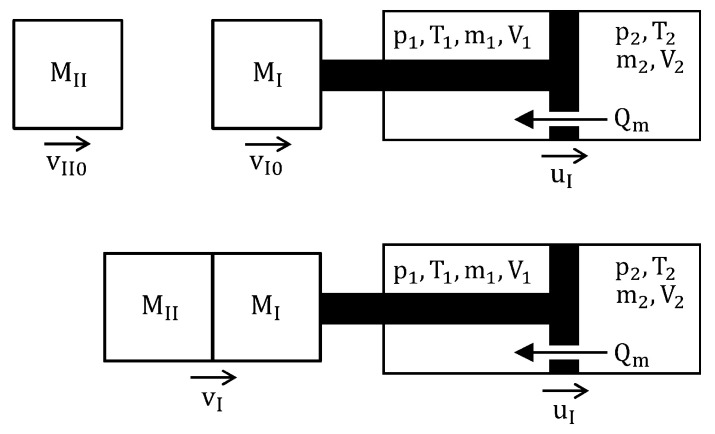
Scheme of the fluid-based absorber under impact excitation—the case of double-impact conditions.

The EPPC method will be discussed using an impact-absorbing system in the form of double-chamber cylinder-piston device (Figure 1). Both chambers of the shock absorber are filled with working medium, which is compressible pneumatic fluid. As a result of applied impact loading caused by the collision of an object or external force, the piston moves forward, causing an increase in pressure in the contracted chamber, a decrease in pressure in the elongated chamber, and flow of the medium through the orifice located inside the piston. The process of energy dissipation is caused by irreversible process of pressure equalization between both chambers.

Semi-active operation of the fluid-based shock absorber can be provided via the application of a sensor system for measurements of the actual thermodynamic state of the gas and the actual kinematics of the piston, as well as via the application of the controllable, fast-operating, electromechanical or piezoelectric valve. The valve controls the actual mass flow rate of the fluid between the chambers, the change of force generated by the absorber, and the corresponding actual deceleration of the impacting object. Thus, fast-operating valves can be effectively applied for real-time management of the impact absorption and energy dissipation process.

As shown in Figure 1, the object of mass ($M_{I})$ and initial velocity ($v_{I0})$ is decelerated due to the reaction force of the absorber transmitted through the piston. During the impact absorption process, a second excitation appears, and it is caused by the contact of the first object with another body of mass ($M_{\mathrm{II}})$. A further part of the impact mitigation process depends on the operation of the absorber as well as the contact conditions between both decelerated objects. Let us consider the most illustrative case of perfectly inelastic collision, when the entire process can be divided into three phases:

- Separate deceleration of the first object;
- Impact of the second object—the first is object under influence of the absorber reaction force and contact force between both decelerated objects;
- Deceleration of joint objects.

In comparison to studies conducted previously by the authors, the impact mitigation problem with unknown disturbance forces [44] is extended to the case of simultaneously unknown disturbance forces and the unknown mass of the impacting object. In order to formulate this problem and discuss a new equivalent parameter predictive control method, the model of the double-chamber shock absorber under double-impact conditions has to be introduced.

### 2.2. State–Space Model of the Considered System

The applied mathematical model of the double-chamber fluid-based shock absorber subjected to impact excitation is based exclusively on fundamental physical principles. In the most general case, the model includes: (i) equations of motion of impacting objects; (ii) two equations of fluid volume (or fluid mass) balance; (iii) two equations of fluid energy balance; (iv) equation of state of the fluid; (v) equation describing fluid flow between the chambers; and (vi) kinematic definitions of chambers’ volumes. The equations of fluid volumes balance are typically used for hydraulic fluids with small compressibility, while the equations of mass balance are used for highly compressible pneumatic fluids.

The state–space representation of the shock absorber model, which involves fluid volume balance, is typically obtained by choosing pressures ($p_{1},p_{2})$ and temperatures of the fluid ($T_{1},T_{2})$ as state variables, eliminating the remaining variables using algebraic equations of the model and solving the resulting balance equations with respect to ${\dot{p}}_{1},{\dot{p}}_{2},{\dot{T}}_{1},\;{\dot{T}}_{2}$. In turn, the state–space representation of the model with fluid mass balance can be obtained by choosing either the masses ($m_{1},m_{2})$ and pressures $\left(p_{1},p_{2}\right)$ or masses and temperatures $\left(m_{1},m_{2},T_{1},T_{2}\right)$ of the fluid as state variables, which leads to a simpler final form of the governing equations.

The exact form of state–space representation depends on the assumed equation of state of the fluid, which is expressed in terms of fluid pressure (p), temperature (T), and density (ρ) in the form $f_{s}\left(p,T,\rho \right)=0$, resulting in definitions of fluid compressibility and thermal expansion coefficients β and α, and the definitions of specific internal energy $\bar{U}$ and specific enthalpy $\bar{H}$. Here, we will consider the explicit form of the mathematical model for the pneumatic double-chamber shock absorber with the ideal gas used as a working fluid. In such a case, the equation of state is defined by the function:

(1) $$f_{s}\left(p,T,\rho \right)=p-\rho \mathrm{RT},$$

which yields:

(2) $$\beta =-\frac{1}{V}\frac{\partial V}{\partial p}=p^{-1},\;\;\;\;\alpha =\frac{1}{V}\frac{\partial V}{\partial T}=T^{-1},$$

(3) $$\bar{U}=\left(c_{p}-\frac{\alpha p}{\rho }\right)T+\left(\beta p-\alpha T\right)\frac{p}{\rho }=c_{v}T,\;\;\;\;\bar{H}=c_{p}T+\left(1-\alpha T\right)\frac{p}{\rho }=c_{p}T,$$

where R is the gas constant, V is volume of the fluid, $c_{p}$ is constant pressure heat capacity, and $c_{v}$ is constant volume heat capacity. The complete physical model of the pneumatic shock absorber is obtained by introducing constitutive Equations (1) and (2) into the general form of balance equations and assuming the proper model of the gas flow.

Herein, the state–space model is presented for the analyzed double-impact scenario, while the shock absorber is considered an adiabatic system without heat transfer through cylinder walls and without delimiting forces confining motion of the piston at the end of the stroke. In such a case, the state–space model utilizes variables $u_{I},v_{I},u_{\mathrm{II}},v_{\mathrm{II}},p_{1},p_{2},T_{1},T_{2}$ and takes the following form:

(4) $$\frac{{\mathrm{du}}_{\mathrm{II}}}{\mathrm{dt}}=v_{\mathrm{II}},$$

(5) $$\frac{{\mathrm{dv}}_{\mathrm{II}}}{\mathrm{dt}}=-M_{\mathrm{II}}^{-1}F_{c}\left(t\right),$$

(6) $$\frac{{\mathrm{du}}_{I}}{\mathrm{dt}}=v_{I},$$

(7) $$\frac{{\mathrm{dv}}_{I}}{\mathrm{dt}}=M_{I}^{-1}\left[F_{\mathrm{ext}}\left(t\right)-F_{\mathrm{dist}}\left(t\right)-F_{p}\left(p_{1},p_{2}\right)+F_{c}\left(t\right)\right],$$

(8) $$\frac{{\mathrm{dp}}_{1}}{\mathrm{dt}}=\frac{\kappa }{V_{1}}\left[-p_{1}{\dot{V}}_{1}+Q_{m}{\mathrm{RT}}_{2}\right],$$

(9) $$\frac{{\mathrm{dp}}_{2}}{\mathrm{dt}}=\frac{\kappa }{V_{2}}\left[-p_{2}{\dot{V}}_{2}-Q_{m}{\mathrm{RT}}_{2}\right],$$

(10) $$\frac{{\mathrm{dT}}_{1}}{\mathrm{dt}}=\frac{{\mathrm{RT}}_{1}}{c_{v}p_{1}V_{1}}\left[Q_{m}\left(c_{p}T_{2}-c_{v}T_{1}\right)-p_{1}{\dot{V}}_{1}\right],$$

(11) $$\frac{{\mathrm{dT}}_{2}}{\mathrm{dt}}=-\frac{{\mathrm{RT}}_{2}}{c_{v}p_{2}V_{2}}\left[Q_{m}{\mathrm{RT}}_{2}+p_{2}{\dot{V}}_{2}\right],$$

(12) $$\mathrm{IC}:{\;u}_{I}\left(0\right)=u_{I0},{\dot{u}}_{I}\left(0\right)=v_{I0},u_{\mathrm{II}}\left(0\right)=u_{\mathrm{II}0},{\dot{u}}_{\mathrm{II}}\left(0\right)=v_{\mathrm{II}0},\;\;p_{1}\left(0\right)=p_{1}^{0},{\;p}_{2}\left(0\right)=p_{2}^{0},{\;T}_{1}\left(0\right)=T_{1}^{0},{\;T}_{2}\left(0\right)=T_{2}^{0}.$$

The chambers’ volumes are linear functions of piston displacement $V_{1}=f_{1}\left(u_{I}\right)$ and $V_{2}=f_{2}\left(u_{I}\right)$, so their time derivatives are functions of piston velocity ${\dot{V}}_{1}=f_{1}\left(v_{I}\right)$ and ${\dot{V}}_{2}=f_{2}\left(v_{I}\right)$, and Equations (4)–(12) define a classical state–space model. The quantity $M_{I}$ is a joint mass of the first impacting object and the piston, and $M_{\mathrm{II}}$ is a mass of the second impacting object. The pneumatic force generated by the absorber is defined as $F_{p}=p_{2}A_{2}-p_{1}A_{1}$, where $p_{2}$ and $p_{1}$ are pressures in compressed and decompressed chambers, $A_{1}$ and $A_{2}$ are the cross-sectional areas of both chambers, $F_{\mathrm{dist}}$ is the disturbance force which includes, e.g., dry friction or viscous damping, $F_{\mathrm{ext}}$ is the external force acting on the first impacting object, and $F_{c}$ is the contact force acting between both objects. The mass outflow rate from the upstream chamber $Q_{m\;}\left(p_{1},p_{2},T_{2},A_{v}\left(t\right)\right)$ depends on the assumed flow model and the time-dependent area of valve opening $A_{v}\left(t\right)$, which is treated as the control variable. The impact excitation is modeled by masses of the impacting objects $M_{I},\;M_{\mathrm{II}}$ and their initial velocities $v_{I0},\;v_{\mathrm{II}0}$. Let us note that Equations (8)–(11) combine the equations of mass balance and energy balance; hence, the physical purity of the general model of shock absorber model is lost.

The space-state model utilizing variables $u_{I},v_{I},u_{\mathrm{II}},v_{\mathrm{II}},m_{1},m_{2},p_{1},p_{2}$ consists of equations of motion (Equations (4)–(7)), equations of fluid mass balance, and equations describing changes of pressures in both absorber chambers:

(13) $${\dot{m}}_{1}=Q_{m\;},$$

(14) $${\dot{m}}_{2}=-Q_{m\;},$$

(15) $$\frac{{\mathrm{dp}}_{1}}{\mathrm{dt}}=\frac{\kappa }{V_{1}}\left[-p_{1}{\dot{V}}_{1}+Q_{m}\frac{p_{2}V_{2}}{m_{2}}\right],$$

(16) $$\frac{{\mathrm{dp}}_{2}}{\mathrm{dt}}=\frac{\kappa }{V_{2}}\left[-p_{2}{\dot{V}}_{2}-Q_{m}\frac{p_{2}V_{2}}{m_{2}}\right],$$

where the mass flow rate is now defined as a function: $Q_{m}\left(u_{I},m_{2},p_{1},p_{2},\;A_{v}\left(t\right)\right)$. In turn, the state–space model utilizing variables $u_{I},v_{I},u_{\mathrm{II}},v_{\mathrm{II}},m_{1},m_{2},T_{1},T_{2}$ consists of equations of motion (Equations (4)–(7)), equations of fluid mass balance (Equations (13) and (14)), and equations describing change of temperatures in both absorber chambers:

(17) $$\frac{{\mathrm{dT}}_{1}}{\mathrm{dt}}=\frac{1}{c_{v}m_{1}}\left[Q_{m}\left(c_{p}T_{2}-c_{v}T_{1}\right)-\frac{m_{1}{\mathrm{RT}}_{1}}{V_{1}}{\dot{V}}_{1}\right],$$

(18) $$\frac{{\mathrm{dT}}_{2}}{\mathrm{dt}}=-\frac{1}{c_{v}m_{2}}\left[Q_{m}{\mathrm{RT}}_{2}+\frac{m_{2}{\mathrm{RT}}_{2}}{V_{2}}{\dot{V}}_{2}\right],$$

where the mass flow rate is defined as a function $:\;Q_{m}\left(u_{I},m_{2},T_{1},T_{2},\;A_{v}\left(t\right)\right)$.

The number of state variables and state equations in the above models can be reduced by transforming selected differential equations into the algebraic ones. First, summation of the equations of fluid mass balance (Equations (13) and (14)) gives the algebraic equation of mass conservation:

(19) $$m_{1}+m_{2}=\mathrm{const}.$$

Second, the equations of energy balance for the upstream chamber (Equations (16) and (18)) can be integrated analytically to obtain the following:

(20a) $$\frac{p_{2}{\left(V_{2}\right)}^{\kappa }}{{\left(m_{2}\right)}^{\kappa }}=\frac{p_{2}^{0}{\left(V_{2}^{0}\right)}^{\kappa }}{{\left(m_{2}^{0}\right)}^{\kappa }},$$

(20b) $$\frac{T_{2}{\left(V_{2}\right)}^{\kappa -1}}{{\left(m_{2}\right)}^{\kappa -1}}=\frac{T_{2}^{0}{\left(V_{2}^{0}\right)}^{\kappa -1}}{{\left(m_{2}^{0}\right)}^{\kappa -1}}.$$

Third, for the special case $F_{\mathrm{ext}}={\;F}_{\mathrm{dist}}=\mathrm{const}$., the summation of the equations of energy balance (Equations (15) and (16) or Equations (17) and (18)) and their time integration and combining them with integrated equations of motion (Equations (4)–(7)) gives algebraic equations of total energy balance in the following forms:

(21a) $$\frac{1}{2}{\mathrm{Mv}}_{I0}^{2}-\frac{1}{2}{\mathrm{Mv}}_{I}^{2}+\left(F_{\mathrm{ext}}-F_{\mathrm{dist}}\right)\left(u_{I}-u_{I0}\right)=\frac{p_{1}V_{1}}{\kappa -1}+\frac{p_{2}V_{2}}{\kappa -1}-\frac{p_{1}^{0}V_{1}^{0}}{\kappa -1}-\frac{p_{2}^{0}V_{2}^{0}}{\kappa -1},$$

(21b) $$\frac{1}{2}{\mathrm{Mv}}_{I0}^{2}-\frac{1}{2}{\mathrm{Mv}}_{I}^{2}+\left(F_{\mathrm{ext}}-F_{\mathrm{dist}}\right)\left(u_{I}-u_{I0}\right)=m_{1}c_{v}T_{1}+m_{2}c_{v}T_{2}-m_{1}^{0}c_{v}T_{1}^{0}-m_{2}^{0}c_{v}T_{2}^{0},$$

where $M=M_{I}$ during the first stage of impact, and $M=M_{I}+M_{\mathrm{II}}$ during the second stage of impact when both impacting objects move together.

Consequently, the state–space model expressed in variables $u_{I},v_{I},u_{\mathrm{II}},v_{\mathrm{II}},m_{1},m_{2},p_{1},p_{2}$ can be reduced to the state–space model with variables $u_{I},v_{I},u_{\mathrm{II}},v_{\mathrm{II}},m_{1}$ using Equation (19) in order to determine $m_{2}$, Equation (20a) to determine $p_{2}$, and Equation (21a) to determine $p_{1}$. Similarly, the state–space models expressed in variables $u_{I},v_{I},u_{\mathrm{II}},v_{\mathrm{II}},m_{1},m_{2},T_{1},T_{2}$ can be reduced to the state–space model with variables $u_{I},v_{I},m_{1}$ by using Equation (20b) to determine $T_{2}$ and Equation (21b) to determine $T_{1}$. Eventually, both approaches lead to the same form of the state–space model, which takes the following form:

(22) $$\frac{{\mathrm{du}}_{\mathrm{II}}}{\mathrm{dt}}=v_{\mathrm{II}},$$

(23) $$\frac{{\mathrm{dv}}_{\mathrm{II}}}{\mathrm{dt}}=-M_{\mathrm{II}}^{-1}F_{c}\left(t\right),$$

(24) $$\frac{{\mathrm{du}}_{I}}{\mathrm{dt}}=v_{I},$$

(25) $$\frac{{\mathrm{dv}}_{I}}{\mathrm{dt}}=M_{I}^{-1}\left[F_{\mathrm{ext}}\left(t\right)-F_{\mathrm{dist}}\left(t\right)-F_{\mathrm{pneu}}\left(u_{I},v_{I},m_{1}\right)+F_{c}\left(t\right)\right],$$

(26) $$\frac{{\mathrm{dm}}_{1}}{\mathrm{dt}}=Q_{m}\left(u_{I},v_{I},m_{1},A_{v}\left(t\right)\right).$$

The function $Q_{m}\left(u_{I},v_{I},m_{1},{\;A}_{v}\left(t\right)\right)$ is typically fairly complicated due to the relatively high complexity of the function defining the mass flow rate of compressible flow in terms of original variables $Q_{m}\left(p_{1},p_{2},T_{2},A_{v}\left(t\right)\right)$ and the relatively complicated dependence between the arguments of both functions. The fundamental state–space model based on Equations (4)–(12) and its equivalent forms utilizing Equations (13)–(16) and Equations (17) and (18) will be used for the simulation of the considered system, while the simplified model based on Equations (22)–(26) (or equivalent) will be used to derive the predictive model used to simulate response of the system at a single control step, when the assumption of constant values of disturbance and external forces is justified.

## 3. Self-Adaptive Impact Mitigation Using Equivalent Parameter Predictive Control

The self-adaptive impact-absorbing systems use the control of valve opening in order to provide dissipation of the entire impact energy and mitigation of the dynamic response, i.e., minimization of generated force and corresponding impacting object deceleration. The concept assumes that selected parameters of the system, a number of excitation parameters and disturbances that may occur during operation (e.g., additional forces or fluid leakages), are unknown to the controller. Despite the lack of preliminary knowledge about the dynamic excitation, the self-adaptive systems are expected to automatically adapt to the actual impact conditions and provide (sub-) optimal and robust dissipation of the impact energy.

The corresponding mathematical formulations of the impact absorption problem are typically based on tracking the actually optimal system path, which is updated during the process and depends on the actual system state (the so-called state-dependent path-tracking problem). Until now, only the particular solution of the variational problem in which the external and disturbance forces are unknown and all system parameters are predefined and constant, have been proposed. In turn, this paper presents a mathematical formulation of, and solution to, the impact absorption problem in which both the mass of the impacting objects and the time-history of external and disturbance forces remain unknown.

### 3.1. Formulation of the Control Problem

The starting point for further considerations is the force-based state-dependent path-tracking problem [41], which includes absorption of the entire impact energy and minimization of global discrepancy between predicted total force generated by the absorber $F_{\mathrm{abs}}\left(A_{v}\left(t\right),t\right)$ and actual optimal value of this force $F_{\mathrm{abs}}^{\mathrm{opt}}\left(t\right)$:

(27) $$\begin{array}{l} \left.{\mathrm{Find}\;A}_{v}\left(t\right)\right|\;\int_{u_{I0}}^{u_{I}\left(T\right)}F_{\mathrm{abs}}{\mathrm{du}}_{I}=E_{\mathrm{imp}}\;\mathrm{and}\;\int_{0}^{T}{\left(F_{\mathrm{abs}}\left(A_{v}\left(t\right),t\right)-F_{\mathrm{abs}}^{\mathrm{opt}}\left(t\right)\right)}^{2}\mathrm{dt}\;\mathrm{is}\;\mathrm{minimal},\\ \mathrm{subject}\;\mathrm{to}:\;\mathrm{model}\;\mathrm{of}\;\mathrm{system}\;\mathrm{dynamics}\;\mathrm{defined}\;\mathrm{by}\;\mathrm{Equations}\;\left(22\right)–\left(26\right). \end{array}$$

The predicted absorber force $F_{\mathrm{abs}}\left(A_{v}\left(t\right),t\right)$ is defined as the sum of predicted pneumatic and disturbance forces:

(28) $$F_{\mathrm{abs}}\left(A_{v}\left(t\right),t\right)=F_{p}\left(A_{v}\left(t\right)\right)+F_{\mathrm{dist}}\left(t\right).$$

The actual optimal reaction force $F_{\mathrm{abs}}^{\mathrm{opt}}\left(t\right)$ is a constant force which allows us to stop the impacting object exactly at the end of absorber stroke d, and it is determined assuming that contact force $F_{c}\left(t\right)$ and external force $F_{\mathrm{ext}}\left(t\right)\;$ remain constant during the remaining part of the process:

(29) $$F_{\mathrm{abs}}^{\mathrm{opt}}\left(t\right)=\frac{M_{I}{\dot{u}}_{I}{\left(t\right)}^{2}}{2\left(d-u_{I}\left(t\right)\right)}+F_{c}\left(t\right)+F_{\mathrm{ext}}\left(t\right).$$

Moreover, T is the time when the system reaches static equilibrium. Using definitions given by Equations (28) and (29) and omitting the condition of energy dissipation in formulation given by Equation (27) (see [44] for the explanation) yields the following:

(30) $$\begin{array}{l} \left.{\mathrm{Find}\;A}_{v}\left(t\right)\;\right|\int_{0}^{T}{\left(F_{p}\left(A_{v}\left(t\right)\right)-\frac{M_{I}{\dot{u}}_{I}{\left(t\right)}^{2}}{2\left(d-u_{I}\left(t\right)\right)}-\left[F_{c}\left(t\right)+F_{\mathrm{ext}}\left(t\right)-F_{\mathrm{dist}}\left(t\right)\right]\right)}^{2}\mathrm{dt}\;\mathrm{is}\;\mathrm{minimal},\\ \mathrm{subject}\;\mathrm{to}:\;\mathrm{model}\;\mathrm{describing}\;\mathrm{system}\;\mathrm{dynamics}\;\mathrm{defined}\;\mathrm{by}\;\mathrm{Equations}\;\left(22\right)–\left(26\right). \end{array}$$

The proposed solution method is based on the transformation of the above global state-dependent path-tracking problem into a series of standard path-tracking problems starting at subsequent time instants. According to the MPC approach for all subsequent control steps, the applied prediction interval is arbitrarily shortened in order to fasten computations. In the case when the prediction interval has the length of a single control step, the control problem solved at each step reads as follows:

(31) $$\begin{array}{l} \left.{\mathrm{Find}\;A}_{v}\left(t\right)\right|\int_{t_{i}}^{t_{i}+\Delta t}{\left(F_{p}\left(A_{v}\left(t\right)\right)-\frac{M_{I}{\dot{u}}_{I}{\left(t_{i}\right)}^{2}}{2\left(d-u_{I}\left(t_{i}\right)\right)}-\left[F_{c}\left(t_{i}\right)+F_{\mathrm{ext}}\left(t_{i}\right)-F_{\mathrm{dist}}\left(t_{i}\right)\right]\right)}^{2}\mathrm{dt}\;\mathrm{is}\;\mathrm{minimal},\\ \mathrm{subject}\;\mathrm{to}:\;\mathrm{model}\;\mathrm{describing}\;\mathrm{system}\;\mathrm{dynamics}\;\mathrm{defined}\;\mathrm{by}\;\mathrm{Equations}\;\left(22\right)–\left(26\right). \end{array}$$

Although the applied control interval has arbitrary length, the above problem is not local in time since it includes optimal values of reaction force for the entire process. Since values of contact, external, and disturbance forces at the actual time instant are not explicitly known or measured, the path-tracking problem has to be further reformulated. 

The current objective is to modify the formulation given by Equation (31) such that it can serve as a basis for the development of the control method, which will be relevant for an unknown value of impacting mass and an unknown change of external and disturbance forces such as HPC, while maintaining the high precision of IPC resulting from the exact solution of the optimal control problem at each control step. In the proposed approach, the impacting object mass is repeatedly identified at the beginning of each control step using the equation of motion (Equation (25)) for the initial time instant of each control step $\;t_{i}$:

(32) $$M_{I}=-\frac{F_{p}\left(t_{i}\right)}{{\ddot{u}}_{I}\left(t_{i}\right)}+\frac{F_{c}\left(t_{i}\right)+F_{\mathrm{ext}}\left(t_{i}\right)-F_{\mathrm{dist}}\left(t_{i}\right)}{{\ddot{u}}_{I}\left(t_{i}\right)}.$$

Let us note that the above identification is an abstract mathematical procedure since the values of contact, external, and disturbance forces at the r.h.s. of Equation (32) are also not known by the control system. Nevertheless, introducing the definition of identified mass into the discretized state-dependent path-tracking problem (Equation (31)) yields exact formulation of the path-tracking problem:

(33) $$\begin{array}{c} \left.{\mathrm{Find}\;A}_{v}\left(t\right)\;\right|\;\int_{t_{i}}^{t_{i}+\Delta t}(F_{p}\left(A_{v}\left(t\right)\right)+\left[\frac{F_{p}\left(t_{i}\right)}{{\ddot{u}}_{I}\left(t_{i}\right)}-\frac{F_{c}\left(t_{i}\right)+F_{\mathrm{ext}}\left(t_{i}\right)-F_{\mathrm{dist}}\left(t_{i}\right)}{{\ddot{u}}_{I}\left(t_{i}\right)}\right]\frac{{\dot{u}}_{I}{\left(t_{i}\right)}^{2}}{2\left(d-u_{I}\left(t_{i}\right)\right)}-\\ {\left[F_{c}\left(t_{i}\right)+F_{\mathrm{ext}}\left(t_{i}\right)-F_{\mathrm{dist}}\left(t_{i}\right)\right])}^{2}\mathrm{dt}\;\mathrm{is}\;\mathrm{minimal} \end{array}$$

The above exact formulation can be transformed into its approximate version:

(34) $$\left.{\;\mathrm{Find}\;A}_{v}\left(t\right)\;\right|\;\int_{t_{i}}^{t_{i}+\Delta t}{\left(F_{p}\left(A_{v}\left(t\right)\right)+\frac{F_{p}\left(t_{i}\right)}{{\ddot{u}}_{I}\left(t_{i}\right)}\frac{{\dot{u}}_{I}{\left(t_{i}\right)}^{2}}{2\left(d-u_{I}\left(t_{i}\right)\right)}\right)}^{2}\mathrm{dt}\;\mathrm{is}\;\mathrm{minimal},$$

when either kinematic or static conditions of equivalence are satisfied. The kinematic condition of equivalence requires that the impacting object deceleration at the beginning of the control step approximately equals the optimal value of constant deceleration for which the object is stopped at the end of absorber stroke:

(35) $$-{\ddot{u}}_{I}\left(t_{i}\right)≅\frac{{\dot{u}}_{I}{\left(t_{i}\right)}^{2}}{2\left(d-u_{I}\left(t_{i}\right)\right)}.$$

In such a case, the sum of contact, external, and disturbance forces, which occurs in the numerator of the third term and in the fourth term of the Equation (33), can be simultaneously eliminated, and transition between both formulations of the control problem is straightforward. In turn, the static conditions of equivalence are fulfilled when one of the following conditions is satisfied:

- The sum of additional forces acting in the system is small in comparison to the pneumatic force;
- The change of the pneumatic force during a single control step is relatively small.

The proof of the above statement is included in the Appendix A. The first condition indicates a trivial case when pneumatic force prevails in system response, which provides direct transition between exact and approximate formulation of the control problem. In turn, the second condition indicates that the equivalence of both formulations requires adequately short control steps, which prevents large changes of pneumatic force. 

The time-dependent term being the multiplier of optimal acceleration in the approximate variational formulation (Equation (34)) can be interpreted as the equivalent mass of the impacting object:

(36) $$M_{\mathrm{eq}}\left(t_{i}\right)=-\frac{F_{p}\left(t_{i}\right)}{{\ddot{u}}_{I}\left(t_{i}\right)}.$$

The values of equivalent mass change in the subsequent control steps and compensate the presence of external and disturbance forces acting on the impacting object. The introduction of the equivalent mass allows us to rewrite Equation (34) as a path-tracking problem in the following form:

(37) $$\left.{\mathrm{Find}\;A}_{v}\left(t\right)\;\right|\;\int_{t_{i}}^{t_{i}+\Delta t}{\left(F_{p}\left(A_{v}\left(t\right)\right)-M_{\mathrm{eq}}\left(t_{i}\right)\frac{{\dot{u}}_{I}{\left(t_{i}\right)}^{2}}{2\left(d-u_{I}\left(t_{i}\right)\right)}\right)}^{2}\mathrm{dt}\;\mathrm{is}\;\mathrm{minimal}.$$

The above version of the path-tracking problem is the basis for development of the EPPC.

According to the above derivation, the variational formulation of the impact mitigation problem given by Equation (37) is an approximate formulation in the case when contact, external, and disturbance forces are assumed constant during the considered control step. It can be also proven that it is an exact formulation in the case when predicted forces are defined by scaling the initial force value by the ratio of actual and initial acceleration:

(38) $$F_{c}\left(t\right)=F_{c}\left(t_{i}\right)\frac{{\ddot{u}}_{I}\left(t\right)}{{\ddot{u}}_{I}\left(t_{I}\right)},{\;F}_{\mathrm{ext}}\left(t\right)=F_{\mathrm{ext}}\left(t_{i}\right)\frac{{\ddot{u}}_{I}\left(t\right)}{{\ddot{u}}_{I}\left(t_{i}\right)},\;{\;F}_{\mathrm{dist}}\left(t\right)=F_{\mathrm{dist}}\left(t_{i}\right)\frac{{\ddot{u}}_{I}\left(t\right)}{{\ddot{u}}_{I}\left(t_{i}\right)}.$$

The above definitions indicate that predicted contact, external, and disturbance forces have to be assumed as forces of the inertial type. Such an assumption is fully justified for the contact force acting between two impacting objects in the considered double-impact scenario, during the stage when the objects move together with the same deceleration, providing $F_{c}\left(t_{i}\right)=-M_{\mathrm{II}}{\ddot{u}}_{I}\left(t_{i}\right)$ and $F_{c}\left(t\right)=-M_{\mathrm{II}}{\ddot{u}}_{I}\left(t\right)$.

### 3.2. Derivation of Equivalent Parameter Predictive Control

The EPPC is a novel control method, in which selected parameters of the system and excitation are identified and updated during the entire impact mitigation process. It can be classified as an adaptive control algorithm since it adapts to unknown or changing parameters of the system. On the other hand, it utilizes a predictive algorithm based on the paradigm of MPC, in which the optimal control problem is solved repeatedly at selected prediction intervals, providing robustness to changes of external excitation and system disturbances.

In particular, the presented implementation of the EPPC has to meet the following requirements concerning operation of the impact absorbing system:

- Automatic adaptation to unknown impacting object mass and its possible changes;
- Automatic adaptation to various initial velocities of the impacting object;
- Adaptation to additional external forces occurring during the process including the case of double-impact excitation;
- Robustness to process disturbances such as unknown friction forces inside the absorber.

Since EPPC is dedicated to the case when both system and excitation parameters are not known, the direct identification of these quantities based on a mathematical model of the system is not possible. Thus, the EPPC is based rather on the determination of equivalent quantities, which substitute system parameters and excitation changes. The application of these equivalent quantities, combined with the repetitive solution of the optimal control problems at subsequent time intervals, ensures precise tracking of the actually optimal system path.

In order to derive the predictive model, which is used for solution of the control problems at subsequent control steps, we proceed analogously to the derivation of the variational formulation (Equation (37)). The impacting object mass is defined by Equation (32), in which it is expressed in terms of known pneumatic force and piston deceleration, as well as unknown contact, external, and disturbance forces. By introducing the above definition into the equation of object motion defined for the considered prediction interval (Equation (25)), we obtain the following equation:

(39) $$\left(-\frac{F_{p}\left(t_{i}\right)}{{\ddot{u}}_{I}\left(t_{i}\right)}+\frac{F_{c}\left(t_{i}\right)+F_{\mathrm{ext}}\left(t_{i}\right)-F_{\mathrm{dist}}\left(t_{i}\right)}{{\ddot{u}}_{I}\left(t_{i}\right)}\right){\ddot{u}}_{I}+F_{p}\left(t\right)=F_{c}\left(t\right)+F_{\mathrm{ext}}\left(t\right)-F_{\mathrm{dist}}\left(t\right).$$

Further, by assuming constant values of contact, external, and disturbance forces, one obtains:

(40) $$\left(-\frac{F_{p}\left(t_{i}\right)}{{\ddot{u}}_{I}\left(t_{i}\right)}+\frac{F_{c}\left(t_{i}\right)+F_{\mathrm{ext}}\left(t_{i}\right)-F_{\mathrm{dist}}\left(t_{i}\right)}{{\ddot{u}}_{I}\left(t_{i}\right)}\right){\ddot{u}}_{I}+F_{p}\left(t\right)=F_{c}\left(t_{i}\right)+F_{\mathrm{ext}}\left(t_{i}\right)-F_{\mathrm{dist}}\left(t_{i}\right).$$

The difference of external and disturbance force which occurs in the second component of the inertial term and at the r.h.s. of the Equation (40) can be simultaneously eliminated, and, consequently, the equation of motion for a single prediction interval can be written in an approximate form:

(41) $$-\frac{F_{p}\left(t_{i}\right)}{{\ddot{u}}_{I}\left(t_{i}\right)}{\ddot{u}}_{I}+F_{p}\left(t\right)=0,$$

if either kinematic conditions of equivalence (Equation (35)) or static conditions of equivalence are satisfied. The proof of the above statement is similar to the proof included in Appendix A.

The quantity occurring in the derived approximate equation of motion (Equation (40)) as a multiplier of acceleration, $-F_{p}\left(t_{i}\right)/\ddot{u}\left(t_{i}\right)$, can be recognized as that introduced in Equation (35) equivalent mass $M_{\mathrm{eq}}\left(t_{i}\right)$, which allows us to rewrite the equation of motion at a single prediction interval in the following form:

(42) $$M_{\mathrm{eq}}\left(t_{i}\right){\ddot{u}}_{I}+F_{p}\left(t\right)=0.$$

The above formula will be further called the equivalent equation of motion. The assumption of the equation of motion cannot be considered as neglecting of the terms denoting contact, disturbance, and external forces, since they are included in the continuously updated time-dependent equivalent mass. The derived equivalent equation of motion is identical to the original equation of motion given by Equation (39) when actual values of contact, external, and disturbance forces are obtained by scaling their initial values by the ratio of actual and initial accelerations (cf. also the comments on exactness of the variational formulation defined by Equation (37):

(43) $$F_{c}\left(t\right)+F_{\mathrm{ext}}\left(t\right)-{\;F}_{\mathrm{dist}}\left(t\right)=\left[F_{c}\left(t_{i}\right)+F_{\mathrm{ext}}\left(t_{i}\right)-F_{\mathrm{dist}}\left(t_{i}\right)\right]\frac{{\ddot{u}}_{I}\left(t\right)}{{\ddot{u}}_{I}\left(t_{i}\right)}.$$

As mentioned, the above scaling is fully justified for the contact force acting in the case of double-impact scenario. In turn, for the external and disturbance forces, it can be used in the vicinity of the optimal system path where ${\ddot{u}}_{I}\left(t\right)≅{\ddot{u}}_{I}\left(t_{i}\right)$ and the difference between forces scaled by the acceleration ratio and constant forces is relatively small.

The proposed methodology allows us to derive a complete predictive model of the considered impact absorbing system, which is used by a controller to simulate and optimize dynamic response at a single prediction interval. The predictive model refers only to the motion of the first impacting object, whereas influence of the second object is included in the recomputed equivalent mass. The state–space version of predictive model is obtained analogously as standard state–space model (Equations (22)–(26)) and includes state equations:

(44) $$\frac{{\mathrm{du}}_{I}}{\mathrm{dt}}=v_{I},$$

(45) $$\frac{{\mathrm{dv}}_{I}}{\mathrm{dt}}=-M_{\mathrm{eq}}{\left(t_{i}\right)}^{-1}F_{p}\left(u_{I},v_{I},m_{1}\right),$$

(46) $$\frac{{\mathrm{dm}}_{1}}{\mathrm{dt}}=Q_{m}\left(u_{I},v_{I},m_{1},A_{v}\left(t\right)\right),$$

(47) $$\mathrm{IC}:u_{I}\left(t_{i}\right)=u_{I0},{\;v}_{I}\left(t_{i}\right)=v_{I0},{\;m}_{1}\left(t_{i}\right)=m_{1}^{0},$$

which are complemented by the following algebraic equations:

(48) $$m_{1}+m_{2}=\mathrm{const}.,$$

(49) $$\frac{p_{2}{\left(V_{2}\right)}^{\kappa }}{{\left(m_{2}\right)}^{\kappa }}=\frac{p_{2}^{0}{\left(V_{2}^{0}\right)}^{\kappa }}{{\left(m_{2}^{0}\right)}^{\kappa }},$$

(50) $$\frac{1}{2}M_{\mathrm{eq}}\left(t_{i}\right)v_{I0}^{2}-\frac{1}{2}M_{\mathrm{eq}}\left(t_{i}\right)v_{I}^{2}=\frac{p_{1}V_{1}}{\kappa -1}+\frac{p_{2}V_{2}}{\kappa -1}-\frac{p_{1}^{0}V_{1}^{0}}{\kappa -1}-\frac{p_{2}^{0}V_{2}^{0}}{\kappa -1},$$

(51) $$f_{s}\left(p_{1},T_{1},\rho_{1}\right)=0,\;f_{s}\left(p_{2},T_{2},\rho_{2}\right)=0,$$

(52) $$V_{1}=V_{1}^{0}+u_{I}A_{1},\;V_{2}=V_{2}^{0}-u_{I}A_{2}.$$

The above predictive model contains the equivalent equation of motion (Equation (45)), which includes exclusively equivalent mass and pneumatic force as well as the global equation of energy balance (Equation (50)) with the l.h.s. expressed exclusively by the equivalent mass and piston velocity. Although the predictive model seems to be simpler than the standard state–space model, it has to be emphasized that the equivalent mass $M_{\mathrm{eq}}$ has to be repeatedly modified at the beginning of each control step depending on the actual measurements of pneumatic force and piston deceleration.

The above predictive model can used within equivalent parameter predictive control for the following purposes:

- Numerical simulation of the system response for arbitrarily assumed change of valve opening via the arbitrary time-integration method—numerical dynamics prediction (NDP);
- Analytical simulation of the system response for selected time-histories of valve opening for which an analytical solution of the predictive model exists—analytical dynamics prediction (ADP).

The proposed implementations of the EPPC include two control strategies. The first one is the optimal control strategy, which utilizes the continuous function describing the change of valve opening during a single prediction interval and direct simulation of system response using NDP:

(53) $$\begin{array}{l} {\mathrm{Find}\;A}_{v}^{\mathrm{opt}}\left(t\right)=\arg\min\int_{t_{i}}^{t_{i}+\Delta t}{\left(F_{p}\left(A_{v}\left(t\right)\right)-M_{\mathrm{eq}}\left(t_{i}\right)\frac{{\dot{u}}_{I}{\left(t_{i}\right)}^{2}}{2\left(d-u_{I}\left(t_{i}\right)\right)}\right)}^{2}\mathrm{dt}\;\\ \mathrm{subject}\;\mathrm{to}:\;\mathrm{state}–\mathrm{space}\;\mathrm{model}\;\mathrm{for}\;a\;\sin\mathrm{gle}\;\mathrm{prediction}\;\mathrm{interval}\;\left(\mathrm{Equations}\;\left(44\right)–\left(52\right)\right),\;\\ \mathrm{where}:{\;A}_{v}\left(t\right)\;\mathrm{is}\;a\;\mathrm{continuous}\;\mathrm{function}\;\mathrm{defining}\;\mathrm{the}\;\mathrm{time}\;\mathrm{course}\;\mathrm{of}\;\mathrm{valve}\;\mathrm{opening} \end{array}$$

The second implementation is the sub-optimal analytical control strategy, which utilizes a selected parameterized function defining the change of valve opening, providing that simulation of system response can be conducted analytically using ADP:

(54) $$\begin{array}{l} \mathrm{Find}\;\beta^{opt}=\arg\min\int_{t_{i}}^{t_{i}+\Delta t}{\left(F_{p}\left(A_{v}\left(\beta ,t\right),t\right)-M_{\mathrm{eq}}\left(t_{i}\right)\frac{{\dot{u}}_{I}{\left(t_{i}\right)}^{2}}{2\left(d-u_{I}\left(t_{i}\right)\right)}\right)}^{2}\mathrm{dt}\\ \mathrm{subject}\;\mathrm{to}:\;\mathrm{state}–\mathrm{space}\;\mathrm{model}\;\mathrm{for}\;a\;\sin\mathrm{gle}\;\mathrm{prediction}\;\mathrm{interval}\;\left(\mathrm{Equations}\;\left(44\right)–\left(52\right)\right),\;\\ \mathrm{where}:\;\beta —{\mathrm{the}\;\mathrm{vector}\;\mathrm{of}\;\mathrm{coefficients}\;\mathrm{of}\;\mathrm{the}\;\mathrm{continuous}\;\mathrm{function}\;A}_{v}\left(\beta ,t\right)\;\mathrm{defining}\;\\ \;\;\;\;\;\;\;\;\;\;\;\;\;\;\;\;\;\mathrm{the}\;\mathrm{change}\;\mathrm{of}\;\mathrm{valve}\;\mathrm{opening}\;\mathrm{at}\;a\;\sin\mathrm{gle}\;\mathrm{prediction}\;\mathrm{interval},\;\mathrm{for}\;\mathrm{which}\;\mathrm{an}\\ \;\;\;\;\;\;\;\;\;\;\;\;\;\;\;\;\;\mathrm{analytical}\;\mathrm{solution}\;\mathrm{of}\;\mathrm{the}\;\mathrm{predictive}\;\mathrm{model}\;\left(\mathrm{Equations}\;\left(44\right)–\left(52\right)\right)\;\mathrm{exists}. \end{array}$$

The following sections will be focused on the second control strategy, being the most promising due to its high computational efficiency, which is crucial for practical implementation of the control systems for impact absorption.

## 4. Equivalent Parameter Predictive Control: Sub-Optimal Analytical Control Strategy

The objective of this section is the detailed description of the implementation of the EPPC based on the sub-optimal analytical control strategy. The presented implementation utilizes the class of functions describing the time course of valve opening, which result in an analytical solution of the predictive model. The advantage of the method is the elimination of the numerical integration of the equations of the predictive model and possibility of the analytical solution to the optimization problem formulated at each control step. Consequently, the method provides similar accuracy for the path-tracking process as the optimal control strategy but with significantly lower computational cost.

### 4.1. Determination of Analytical Functions Defining Change of Valve Opening

The crucial point of the sub-optimal analytical control strategy is the determination of functions of valve opening $A_{v}\left(t\right)={\tilde{A}}_{v}\left(t\right)$, for which a closed analytical solution of the predictive model defined by Equations (44)–(52) exists. Unfortunately, the direct analytical solution of the predictive model for $A_{v}\left(t\right)$, obtained with the use of computer algebra systems, involves complicated time integrals of $A_{v}\left(t\right)$ and does not allow us to identify the cases when the analytical solution can be found. Thus, specific functions ${\tilde{A}}_{v}\left(t\right)$ can possibly be determined by assuming that the evolution of arbitrary components of the system response, chosen between $p_{i}\left(t\right)$, $m_{i}\left(t\right)$, $T_{i}\left(t\right)$, $u_{I}\left(t\right)$, $v_{I}\left(t\right)$, and $a_{I}\left(t\right),\;$ is given by a known function of time, and by solving the resulting system of equations (with respect to $A_{v}\left(t\right)$).

In the considered case, the assumption of quantity $p_{i}\left(t\right)$, $m_{i}\left(t\right)$, or $T_{i}\left(t\right)$ leads to the problem involving differential equations with respect to $u_{I}\left(t\right)$, which has no general analytical solution. In turn, the assumption of known evolution of pneumatic force $F_{p}\left(t\right)=A_{2}p_{2}-A_{1}p_{1}$ and corresponding impacting object deceleration $a_{I}\left(t\right)$, whose proportionality is expressed by equivalent mass $M_{\mathrm{eq}}$, leads to a system with eight algebraic equations:

(55) $$a_{I}\left(t\right)=-M_{\mathrm{eq}}{}^{-1}\left(A_{2}p_{2}-A_{1}p_{1}\right),$$

(56) $$m_{1}+m_{2}=\mathrm{const}.,$$

(57) $$\frac{p_{2}{\left(V_{2}\right)}^{\kappa }}{{\left(m_{2}\right)}^{\kappa }}=\frac{p_{2}^{0}{\left(V_{2}^{0}\right)}^{\kappa }}{{\left(m_{2}^{0}\right)}^{\kappa }},$$

(58) $$\frac{1}{2}M_{\mathrm{eq}}v_{I0}^{2}-\frac{1}{2}M_{\mathrm{eq}}v_{I}^{2}=\frac{p_{1}V_{1}}{\kappa -1}+\frac{p_{2}V_{2}}{\kappa -1}-\frac{p_{1}^{0}V_{1}^{0}}{\kappa -1}-\frac{p_{2}^{0}V_{2}^{0}}{\kappa -1},$$

(59) $$f_{s}\left(p_{1},T_{1},\rho_{1}\right)=0,\;f_{s}\left(p_{2},T_{2},\rho_{2}\right)=0,$$

(60) $$V_{1}=V_{1}^{0}+u_{I}A_{1},\;V_{2}=V_{2}^{0}-u_{I}A_{2},$$

which can be combined with two standard integral relations joining kinematic quantities:

(61) $$v_{I}=v_{I}\left(t_{i}\right)+\int_{t_{i}}^{t}a_{I}\left(t\right)\mathrm{dt},$$

(62) $$u_{I}=u_{I}\left(t_{i}\right)+\int_{t_{i}}^{t}v_{I}\left(t_{i}\right)\mathrm{dt}+\int_{t_{i}}^{t}\int_{t_{i}}^{t}a_{I}\left(t\right){\mathrm{dt}}^{2}\;,$$

and which allows us to determine analytical functions $p_{i}\left(t\right)$, $m_{i}\left(t\right)$, $T_{i}\left(t\right)$, $u_{I}\left(t\right)$ and $v_{I}\left(t\right)$ defining the complete response of the considered impact absorbing system. In such case, the mass flow rate definition (Equation (46)) enables the finding of the required time-history of valve opening:

(63) $${\tilde{A}}_{v}\left(t\right)=\frac{{\mathrm{dm}}_{1}}{\mathrm{dt}}{\left[{\bar{Q}}_{m}\right]}^{-1},$$

where ${\bar{Q}}_{m}$ denotes mass flow rate through the valve of unitary area. The obtained valve opening depends on the assumed pneumatic force, its time derivative, equivalent mass, initial volumes, and pressures of gas in both chambers and time:

(64) $${\tilde{A}}_{v}\left(t\right)={\tilde{A}}_{v}\left(F_{p}\left(t\right),\frac{{\mathrm{dF}}_{p}\left(t\right)}{\mathrm{dt}},\int_{t_{i}}^{t}F_{p}\left(t\right)\mathrm{dt},\;\int_{t_{i}}^{t}\int_{t_{i}}^{t}F_{p}\left(t\right){\mathrm{dt}}^{2},M_{\mathrm{eq}},p_{1}^{0},p_{2}^{0},{\;V}_{1}^{0},{\;V}_{2}^{0},t\right)$$

and can be alternatively expressed with the use of piston kinematics in the following form:

(65) $${\tilde{A}}_{v}={\tilde{A}}_{v}\left(a_{I}\left(t\right),\frac{{\mathrm{da}}_{I}\left(t\right)}{\mathrm{dt}},v_{I}\left(t\right),u_{I}\left(t\right),M_{\mathrm{eq}},p_{1}^{0},p_{2}^{0},{\;V}_{1}^{0},V_{2}^{0},t\right).$$

The untypical dependence of time-history of valve opening on time derivatives $\frac{{\mathrm{dF}}_{p}}{\mathrm{dt}}$ or $\frac{{\mathrm{da}}_{I}}{\mathrm{dt}}$ results from the occurrence of mass derivative $\frac{{\mathrm{dm}}_{1}}{\mathrm{dt}}$ in its basic definition given by Equation (63). The exact forms of Equations (64) and (65) are unfortunately too long to be presented in the paper. The case when the function defining change of pneumatic force $F_{p}$ is parametrized by a vector β, i.e.,

(66) $$F_{p}=F_{p}\left(\beta ,t\right)$$

and providing that time derivatives and integrals of function $F_{p}$ can be calculated analytically, one obtains the function describing the required valve opening in the following form:

(67) $${\tilde{A}}_{v}={\tilde{A}}_{v}\left(\beta ,M_{\mathrm{eq}},p_{1}^{0},p_{2}^{0},{\;V}_{1}^{0},V_{2}^{0},t\right).$$

Since there exists analytical solution of the inverse problem aimed at finding valve opening required to obtain the assumed change of pneumatic force given by Equation (66), there also exists an analytical solution of the straightforward problem of finding system response resulting from valve opening defined by Equation (66). Thus, the assumption of the proper form of the parametrized function defining the change of valve opening at time ${\tilde{A}}_{v}\left(\beta ,t\right)$ allows us to apply the procedure of analytical dynamics prediction, providing fast and exact analytical simulation of the system response for vector β.

### 4.2. Analytical Solution of the Path-Tracking Problem

The procedure of finding corelated analytical functions describing the change of valve opening ${\tilde{A}}_{v}\left(\beta ,t\right)$ and pneumatic force $F_{p}\left(\beta ,t\right)$ significantly facilitates the solution to the path-tracking problem, which is considered at each prediction interval. Problem simplification results from the fact that the integrand of the minimized functional depends analytically on vector β and, moreover, constraints imposed on valve opening can be transformed into constraints imposed on β. In particular, constraining β can be used to confine the range of valve opening and its operation speed or provide continuity of valve opening between the control steps. Thus, the original variational problem defined for each control step is transformed into the problem of searching for optimal components of vector β and takes the following form:

(68) $$\begin{array}{l} \mathrm{Find}\;\beta^{opt}=\arg\min\int_{t_{i}}^{t_{i}+\Delta t}{\left(F_{p}\left(\beta ,t\right)-M_{\mathrm{eq}}\left(t_{i}\right)\frac{{\dot{u}}_{I}{\left(t_{i}\right)}^{2}}{2\left(d-u_{I}\left(t_{i}\right)\right)}\right)}^{2}\mathrm{dt}\\ \mathrm{with}\;\mathrm{respect}\;\mathrm{to}:\;\beta —{\mathrm{vector}\;\mathrm{of}\;\mathrm{coefficients}\;\mathrm{of}\;\mathrm{the}\;\mathrm{continuous}\;\mathrm{function}\;\tilde{A}}_{v}\left(\beta ,t\right)\\ \;\;\;\;\;\;\;\;\;\;\;\;\;\;\;\;\;\;\;\;\;\;\;\;\;\;\;\;\;\;\;\;\mathrm{obtained}\;\mathrm{from}\;\mathrm{the}\;\mathrm{predictive}\;\mathrm{model}\;\left(\mathrm{Equations}\;\left(44\right)–\left(52\right)\right)\\ \;\;\;\;\;\;\;\;\;\;\;\;\;\;\;\;\;\;\;\;\;\;\;\;\;\;\;\;\;\;\;\;{\mathrm{with}\;\mathrm{arbitrary}\;\mathrm{assumed}\;\mathrm{function}\;F}_{p}\left(\beta ,t\right),\\ \mathrm{subject}\;\mathrm{to}:{\;A}_{v}^{\min}\leq {\tilde{A}}_{v}\left(\beta ,t\right)\leq A_{v}^{\max},\;V_{A_{v}}^{\min}\leq \frac{{d\tilde{A}}_{v}\left(\beta ,t\right)}{\mathrm{dt}}\leq V_{A_{v}}^{\max}. \end{array}$$

Since $A_{v}\left(\beta ,t\right)$ is obtained from the assumed function $F_{p}\left(\beta ,t\right)$ by using the predictive model of the system, the equations of the predictive model are directly incorporated into the optimization problem and do not have to be considered as additional constraining equations.

The solution of the optimization problem given by Equation (4.14) is straightforward when the constraints on valve operation are not considered and the time integral of the function $F_{p}\left(\beta ,t\right)$ can be calculated analytically. The convexity of the obtained function of β can be easily checked, and the problem can be solved using standard optimization methods. In particular, it can be shown that in the simplest case of linear function $F_{p}\left(\beta ,t\right)=F_{p}\left(t_{i}\right)+\beta t$ parameterized by a single directional coefficient, the method provides convergence of the system response to the optimal values.

The solution to the constrained optimization problem is more complicated since the constraints imposed on minimal and maximal valve opening, as well as the speed of valve operation, have to be imposed on vector β. In particular, the extreme values of valve opening can be achieved either at the begin or the end of the considered control step, or at the time instants when time derivative of ${\tilde{A}}_{v}\left(\beta ,t\right)$ equals zero. In the former case, the constraints imposed on ${\tilde{A}}_{v}\left(\beta ,t\right)$ can be directly transformed into constraints imposed on β in the following form:

(69) $$A_{v}^{\min}\leq {\tilde{A}}_{v}\left(\beta ,t_{i}\right)\leq A_{v}^{\max},$$

(70) $$A_{v}^{\min}\leq {\tilde{A}}_{v}\left(\beta ,t_{i}+\Delta t\right)\leq A_{v}^{\max}.$$

In contrast, if the extreme valve opening occurs inside the considered interval, the transformation of the constraint has to be preceded by computation of time $t_{\mathrm{ext}}\left(\beta \right)$ when the extremum is achieved by using the standard condition:

(71) $$t_{\mathrm{ext}}\left(\beta \right)=\left.t\left(\beta \right)\;\right|\;\frac{{d\tilde{A}}_{v}\left(\beta ,t\right)}{\mathrm{dt}}=0.$$

Zero value of computed derivative with respect to time has to be found analytically in order to determine time instant $t_{\mathrm{ext}}$ in terms of vector β. Providing that such procedure can be conducted, the constraints imposed on vector β can be formulated as follows:

(72) $$A_{v}^{\min}\leq {\tilde{A}}_{v}\left(\beta ,t_{\mathrm{ext}}\left(\beta \right)\right)\leq A_{v}^{\max}\;.$$

The constraints given by Equation (72) often do not have to be applied since the requirement of fast computations implies relatively short control intervals and reaching the extrema of valve opening at their initial or final time instants. A similar procedure can be applied to transform constraints imposed on speed of valve opening into constraints imposed on time derivatives of vector β.

The resulting minimization problem with constraints imposed on vector β and its time derivatives can be solved in a classical manner by incorporating constraints into the objective function with the use of Lagrange multipliers and by applying Karush–Kuhn–Tucker conditions to find the minimum.

### 4.3. The Control Algorithm

**Figure 2:**
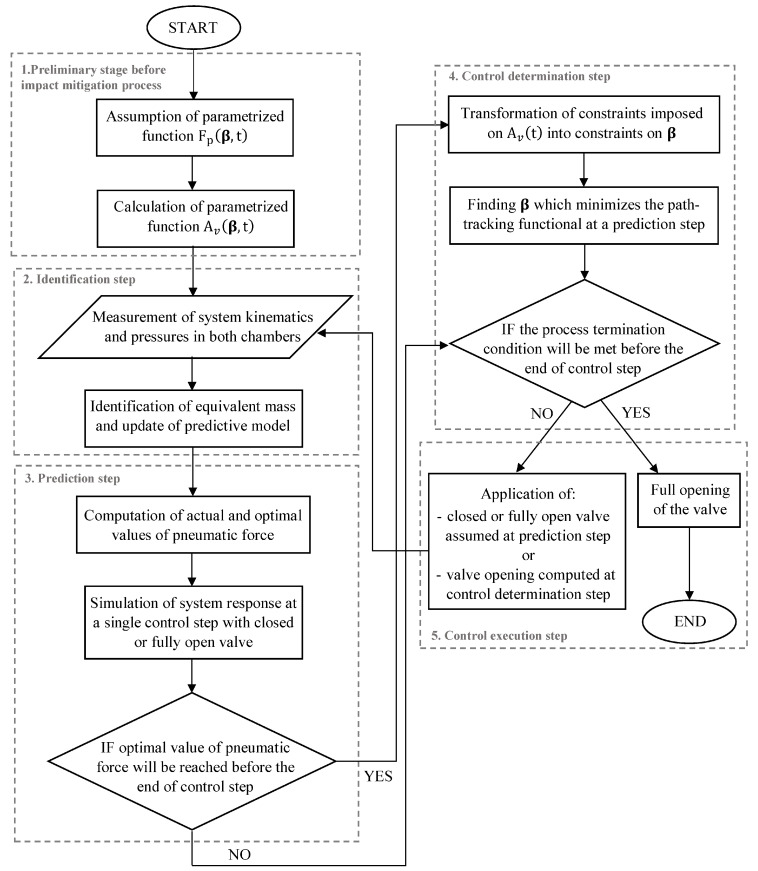
Algorithm applied for the EPPC implementation based on the sub-optimal analytical control strategy.

The numerical implementation of the method described above has been summarized in the form of the process scheme in Figure 2. The algorithm of the EPPC with the sub-optimal analytical control strategy includes the preliminary stage executed before the impact mitigation, in which the pneumatic force is assumed as the parametric function, and the corresponding valve opening function is determined. Further, the impact mitigation process is conducted in four consecutive steps:

- Identification step aimed at the measurement of actual system kinematics and values of pressures in absorber chambers, followed by identification of the equivalent mass parameter used to update the predictive model of the system;
- Prediction step including comparison of actual and optimal values of pneumatic force, and simulation of the system response with extreme valve opening in order to determine if optimal pneumatic force will be reached before the end of control step— if yes, the system starts control determination step; if not, it moves to process termination block;
- Control determination step in which constraints imposed on valve opening are transformed into constraints on vector β; optimization over vector β is conducted in order to minimize path-tracking error at the actual prediction step, and termination condition is checked;
- The control execution step, which depends on the termination condition; if it is not met, the valve opening computed in the control determination step is applied at the actual control step and the system comes back to the identification step; otherwise, the full opening of the valve is applied, and the impact mitigation process is ended.

## 5. Numerical Verification of Equivalent Parameter Predictive Control

The effectiveness of the EPPC was tested using a mathematical model of a double-chamber pneumatic absorber subjected to a standard single-impact excitation and a double-impact excitation involving two objects of unknown masses and initial velocities. In both cases, two types of unknown disturbance forces were considered.

### 5.1. Single-Impact Scenario

The single-impact scenario is the excitation condition wherein the absorber is subjected to the impact of a single object of unknown mass and initial velocity. In such a case, the equations governing motion of mass $M_{\mathrm{II}}$ are not present in the mathematical model of the system, while all other equations remain valid. In order to facilitate comparison with already-developed control strategies, presented numerical examples utilize identical values of the system’s physical parameters, as in previous papers by the authors (see Table 2). The disturbances are caused by elastic and viscous forces characterized by the stiffness coefficient of the linear spring k = 2000 N/m and the damping coefficient of the viscous damper c = 40 Ns/m.

**Figure 3:**
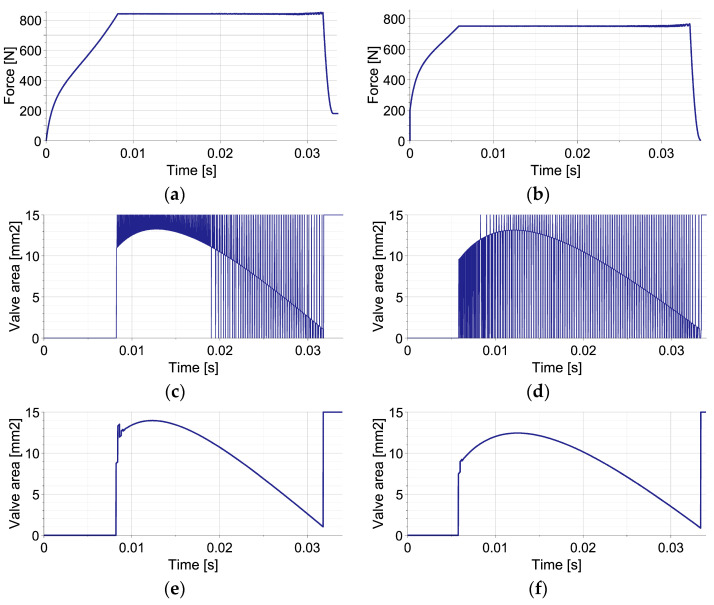
Mitigation of single impact using the EPPC with optimal and sub-optimal analytical control strategies: (**a**,**b**) reaction force in the case of elastic and viscous disturbance for both strategies; (**c**,**d**) valve opening area in the case of elastic and viscous disturbance for the optimal strategy; (**e**,**f**) valve opening area in the case of elastic and viscous disturbance for the sub-optimal analytical strategy.

The EPPC is applied using the optimal control strategy and suboptimal analytical control strategy based on the linear function describing the change of pneumatic force at each control step. The results presented in Figure 3 concern a disturbance by an unknown elastic force (left column) and a disturbance by unknown viscous force (right column). The applied control includes the first stage, when the valve is closed in order to increase generated force to the value required to absorb the entire kinetic energy, and the second stage, when the force is maintained approximately constant.

The comparison of optimal control strategy based on Equation (53) and suboptimal analytical control strategy based on Equation (54) reveals that the obtained approximately constant level of generated force is almost identical in both cases (overlapping lines in Figure 3a,b). The comparison of the required changes of valve opening shows that the optimal control strategy requires intensive control actions with commutative opening and closing of the valve (Figure 3c,d). In contrast, in the suboptimal analytical control strategy, after the initial stage with closed valve, the control remains smooth during the entire period of impact (Figure 3e,f).

The different change of valve opening results from operating principles of both strategies. In the applied implementation of the optimal control strategy at each control step, the valve is initially fully opened/closed in order to compensate for the occurrence of disturbance and obtain the actually optimal value of generated force. Further, the valve opening is continuously modified in order to maintain constant force level. As a result, the discontinuity of valve opening occurs from the time instant when the optimal force level is reached. In contrast, at each control step of the sub-optimal analytical control strategy, the value of parameter β, describing continuous change of valve opening $A_{v}\left(\beta ,t\right)$, is optimized in order to minimize the discrepancy between the predicted and actually optimal value of generated force. Consequently, the change of valve opening at each control step always remains smooth, and relatively small changes between control steps are obtained. During the entire impact period, the valve opening changes gradually and achieves its maximum in the middle of the process.

**Figure 4:**
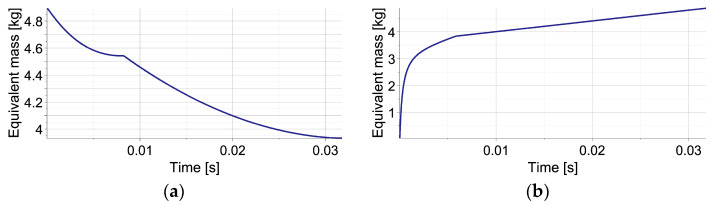
Change of equivalent mass during the considered single-impact scenario with unknown disturbances: (**a**) by elastic force; (**b**) by viscous force.

The change of equivalent mass $M_{\mathrm{eq}}$ during the process can be explained by combining basic definition (Equation (35)) with equation of motion of impacting object (Equations (24) and (25)), which yields the following:

(73) $$M_{\mathrm{eq}}=M_{I}+\frac{F_{\mathrm{dist}}\left(t_{i}\right)}{{\ddot{u}}_{I}\left(t_{i}\right)}=M_{I}\left(1-\frac{F_{\mathrm{dist}}\left(t_{i}\right)}{F_{\mathrm{total}}\left(t_{i}\right)}\right)$$

and reveals explicit dependence on disturbance force $F_{\mathrm{dist}}\;$ and total generated force $F_{\mathrm{total}}$. Consequently, for both types of disturbances, the time history of equivalent mass is substantially different (Figure 4). In the case of elastic disturbance (Figure 4a), the initial value of equivalent mass equals approximately the real mass value (5 kg) since $F_{\mathrm{dist}}≅F_{\mathrm{total}}=0$ and $F_{\mathrm{total}}>F_{\mathrm{dist}}$. During the first stage of impact, when the valve is closed, the nonlinear decrease in $M_{\mathrm{eq}}\left(u_{I}\right)$ results from analytical definitions of $F_{\mathrm{dist}}\left(u_{I}\right)$ and $F_{\mathrm{total}}\left(u_{I}\right)$ substituted into Equation (73) and corresponds to the nonlinear decrease in $M_{\mathrm{eq}}\left(t\right)$ obtained using numerically computed $u_{I}\left(t\right)$. During the second stage, when the total force is maintained constant, $M_{\mathrm{eq}}\left(u_{I}\right)$ decreases linearly due to the linear change of $F_{\mathrm{dist}}\left(u_{I}\right)$; thus, $M_{\mathrm{eq}}\left(t\right)$ decreases as a quadratic function. In turn, in the case of viscous disturbance (Figure 4b), the initial value of the equivalent mass equals approximately zero since $F_{\mathrm{dist}}≅F_{\mathrm{total}}$. Equation (73) allows us to conclude the nonlinear increase in $M_{\mathrm{eq}}\left(u_{I}\right)$ and $M_{\mathrm{eq}}\left(t\right)$ during the first stage of impact and the linear increase in $M_{\mathrm{eq}}\left(u_{I}\right)$ and $M_{\mathrm{eq}}\left(t\right)$ during the second stage. The final value of equivalent mass equals the real mass (5 kg) since $F_{\mathrm{dist}}=0$.

In the case of the single-impact scenario, the EPPC with the sub-optimal analytical control strategy fulfils all requirements for self-adaptive impact mitigation systems; i.e., is provides absorption of the entire energy of unknown impact with unknown disturbances and ensures a minimal level of total generated force. It has very similar efficiency to the EPPC with the optimal control strategy but eliminates its fundamental disadvantages, including intensive control actions with commutative opening and closing of the valve. During the main part of the impact mitigation process, the valve opening changes smoothly, which indicates control feasibility and its low cost.

### 5.2. Double-Impact Scenario with Various Excitations

The double-impact scenario concerns the excitation condition when the impact of the second object occurs during the process of mitigating the impact of the first object. The masses and initial velocities of both objects are assumed to be unknown and can be significantly different. Moreover, the collision between objects is assumed to be highly inelastic and to result in their joint movement towards the absorber bottom. The control is initially aimed at the optimal mitigation of the first object impact (system does not have information about the second excitation) and further at the mitigation of the joint impact of both objects.

The conducted numerical simulations utilize the full mathematical model of the double-chamber pneumatic absorber based on Equations (4)–(12) in order to compute the system response. The system parameters are identical to those of the single-impact scenario in Section 5.1, while the parameters of both impacts are collected in Table 3. The definition of the contact force acting between both objects includes a stiffness term and a mixed stiffness-damping term with a relatively large damping coefficient, so the collision resembles a perfectly inelastic one. At this stage, disturbance forces are neglected, and the analysis is aimed at investigating the influence of the impact’s timing.

**Figure 5:**
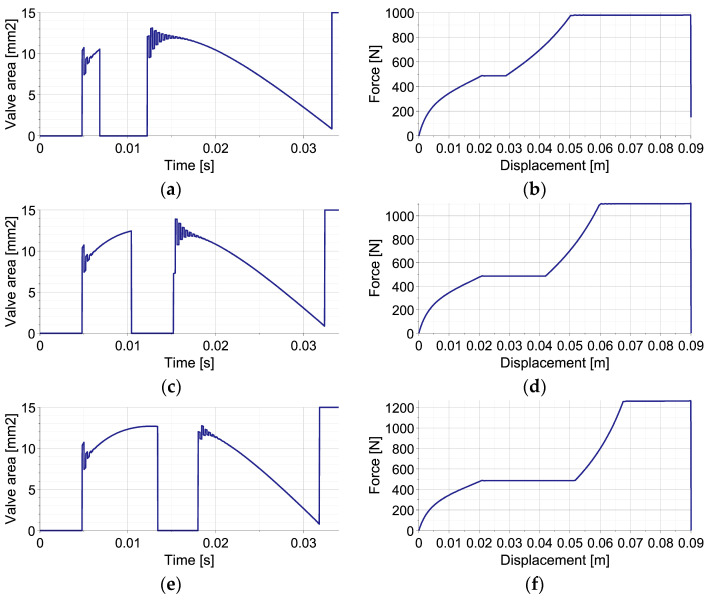
Mitigation of double-impact using EPPC (perfectly inelastic collision): (**a**,**b**) valve opening area and generated reaction force for collisions interval t = 0.0068 s; (**c**,**d**) valve opening area and generated reaction force for collisions interval t = 0.0104 s; (**e**,**f**) valve opening area and generated reaction force for collisions interval t = 0.0134 s.

The results of the numerical simulations of the operation of EPPC with the sub-optimal analytical control strategy for the double-impact scenario with three different times of second impact occurrence are presented in Figure 5. The left column shows the change of applied valve opening in time, while the right one shows the change of force generated by the absorber in terms of first object displacement.

In all cases, at the beginning of the process, the valve remains closed in order to obtain the level of pneumatic force required to absorb the entire kinetic energy of the first object and stop it from using the available part of the absorber’s stroke. Once the optimal pneumatic force is reached, the change of valve opening is optimized at each control step in order to maintain an approximately constant level of generated force. When the impact of the second object occurs, the valve becomes closed in order to increase the pneumatic force to the level required to absorb the joint kinetic energy of both objects. Once the optimal force is reached, the valve opening is again optimized at each control step, and the value of generated pneumatic force is kept approximately constant until the full stroke is reached and both objects are stopped. At the end of the stroke, the valve is fully opened in order to obtain static equilibrium of the system.

Three considered control scenarios are characterized by different valve areas at the end of the first stage of impact, different times of the second valve closing and its re-opening, as well as different changes of valve area during the second stage of impact (Figure 5a,c,e). Consequently, the resulting final level of pneumatic force and the entire force–displacement characteristics are, in both cases, significantly different. Specifically, in the case when the second impact occurs briefly after the first one (Figure 5b), the lowest level of pneumatic force is required during the final stage of impact since a large part of the stroke remains available for the absorption of the actual kinetic energy of both objects. In contrast, in the case when the second impact occurs relatively long after the second one, the highest level of pneumatic force is required since only a small part of the stroke is still available for the absorption of the remaining impact energy (Figure 5f). Let us also note that in all three cases, the rate of pneumatic force increase after the second impact is substantially different, which results from the characteristics of the considered pneumatic dampers. Nevertheless, in all presented cases, the absorbed impact energy (represented by the area below the force–displacement curve) approximately equals the sum of the kinetic energies of both impacting objects, while small discrepancies result only from different amounts of energy absorbed and dissipated at the contact interface.

The example reveals that the control actions required at the beginning of the process for maintaining constant pneumatic force include significantly larger oscillations of valve opening (Figure 5a,c,e) than in the case of single-impact scenario (Figure 3e,f), which is caused by the lack of additional elastic or viscous forces in the system. Moreover, after the second impact, such valve opening oscillations last longer due to large amplitudes and rapid changes of the contact force generated at the contact interface.

**Figure 6:**
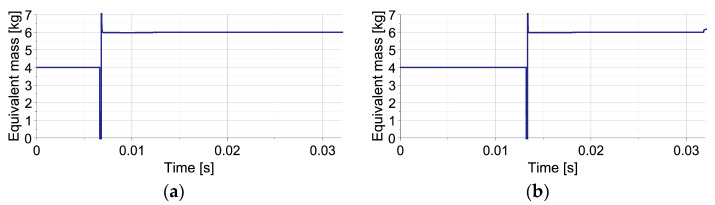
Change of equivalent mass during two double-impact scenarios with the shortest and the longest considered times of the second impact’s occurrence: (**a**) t = 0.0068 s; (**b**) t = 0.0134 s.

The plots of equivalent mass $M_{\mathrm{eq}}$ are presented in Figure 6 for the shortest and the longest considered times of the second impact occurrence. In the analyzed no-disturbance case, during the first impact, the equivalent mass initially equals the mass of the first object ($M_{I})$. During the impact of the second object, when the acceleration of the first object changes its sign and crosses the zero value twice, the equivalent mass temporarily achieves minus and plus infinity (cf. Equation (73)). Finally, during mitigation of joint impact of both objects the equivalent mass, $M_{\mathrm{eq}}$ equals the sum of their mases $M_{I}+M_{\mathrm{II}}$. In this case, the equivalent mass denotes the real value of the total impacting mass since no disturbances are present in the system.

Further analyses are focused on the influence of the non-perfectly inelastic collision of both impacting objects on the operation of the control system. Consequently, numerical simulations are conducted with significantly decreased values of the damping coefficient of the contact interface, which mean that the kinematics of both objects after the collision were not identical.

**Figure 7:**
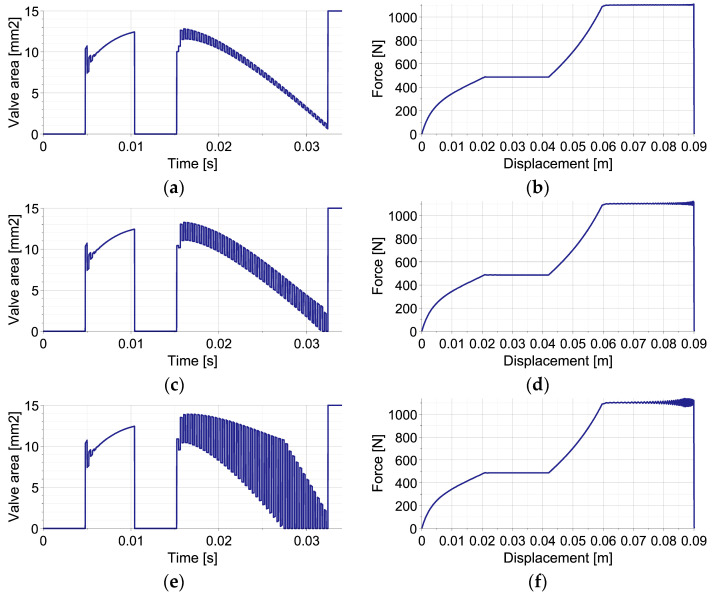
Mitigation of double-impact using EPPC (non-perfectly inelastic collision): (**a**,**b**) valve opening area and generated reaction force in the case of a high damping coefficient; (**c**,**d**) valve opening area and generated reaction force in the case of an intermediate damping coefficient; (**e**,**f**) valve opening area and generated reaction force in the case of a low damping coefficient.

The obtained numerical results presented in Figure 7 indicate that partially inelastic collision requires more intensive control actions with jumps of valve opening between the control steps are required in order to maintain a constant pneumatic force level. In particular, for the first considered value of the damping coefficient, the gradually declining oscillations of valve opening last for the entire second stage of impact (Figure 7a), but the value of the generated pneumatic force remains approximately constant (Figure 7b). In the second example, the value of the damping coefficient is selected in such a way that oscillations of valve opening remain similar during the entire second stage of impact (Figure 7c). In such a case, the pneumatic force includes slightly larger oscillations, which increase only at the very end of the process (Figure 7d). Finally, for the smallest applied damping coefficient, the amplitude of valve opening oscillations increases during the first part of the second stage of the process and remains large until its end (Figure 7e). In this case, the corresponding oscillations of pneumatic force at the end of the process are increased, and the maximal value of generated force is larger than in the previous cases (Figure 7f). Nevertheless, the entire impact energy is still dissipated, and both objects are stopped at the end of absorber’s stroke, as required.

The presented examples prove that the operation of the EPPC with the sub-optimal analytical control strategy is fully satisfactory in the case of the double-impact scenario. Despite the lack of knowledge of impact parameters, it provides high-performance absorption of the kinetic energy of both objects with the minimal level of generated pneumatic force and the correspondingly minimal level of impacting object decelerations. Moreover, the EPPC operates correctly when collision is not perfectly inelastic but requires more rapid control actions, which are harder to be executed in practice. Therefore, the design of the proposed adaptive impact mitigation system should provide perfectly inelastic, e.g., plastic, collision between the objects.

### 5.3. Double-Impact Scenario with Various Disturbances

The following analyses are aimed at investigating the influence of two different types of disturbances arising in the system on the adaptation process and impact mitigation effectiveness. The elastic and viscous disturbance forces modeled by stiffness and damping coefficients of the same values, as in the case of single impact scenario, are introduced to the system. Two presented numerical examples concern a double-impact scenario with perfectly inelastic collision and a double-impact scenario with non-perfectly inelastic collision. As it will be proved, in the latter case, the application of additional elastic or viscous elements inside the pneumatic absorber provides stabilization of the applied control process and system response.

**Figure 8:**
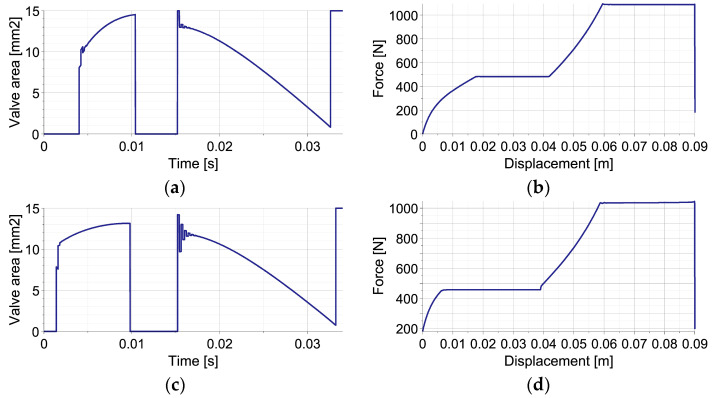
Mitigation of double-impact with disturbance using EPPC (perfectly inelastic collision): (**a**,**b**) valve opening area and generated reaction force in the case of elastic disturbance; (**c**,**d**) valve opening area and generated reaction force in the case of viscous disturbance.

The first numerical example assumes a relatively high value of the damping coefficient of the contact interface, which results in perfectly inelastic collision between colliding objects. The case of the double-impact scenario with elastic disturbance force is presented in Figure 8. At first, it can be observed that despite the presence of the disturbance force, the controller successfully maintains constant levels of the total generated force, the entire impact energy is absorbed, and the objects are stopped using the entire stroke of the absorber. Secondly, it can be seen that the oscillations of valve opening at the beginning of the first and the second impact are smaller than in the case without disturbance (Figure 8a vs. Figure 5c).

Similar analysis was conducted for the double-impact scenario with a viscous disturbance force. The conducted simulations confirm the results obtained previously for the elastic disturbance. In particular, the presence of a viscous disturbance does not violate the operation of the proposed control strategy, the total generated force is effectively maintained constant, and objects are stopped at the end of absorber stroke. Moreover, the oscillations of valve opening at the beginning of both impacts are smaller than in case without disturbance (Figure 8c vs. Figure 5c). Let us also note that the values of generated forces differ slightly between each other and in comparison to no-disturbance cases depending on the values of the applied elastic and viscous coefficients.

**Figure 9:**
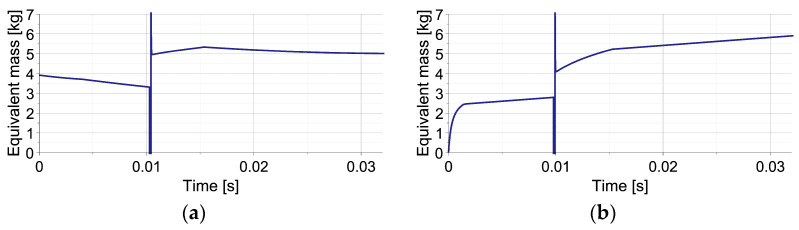
The change of equivalent mass during double-impact scenarios presented in Figure 8: (**a**) elastic disturbance force; (**b**) viscous disturbance force.

Another considered aspect is the change of equivalent mass ($M_{\mathrm{eq}})$ during the process (Figure 9). In the case of elastic disturbance, during the first impact, $M_{\mathrm{eq}}$ decreases analogously to the case of single-impact scenario (cf. Figure 4a). In turn, at the beginning of the second impact, its value temporarily reaches minus and plus infinity. Further, it is affected by both the contact and disturbance forces and changes non-monotonically with a maximum at the time instant, indicating the start of maintaining constant reaction force. In the case of viscous disturbance, the change of $M_{\mathrm{eq}}$ during the first impact is also similar as in case of single-impact (cf. Figure 4b). However, after the temporal reaching of the infinite values at the beginning of the second impact, it increases with different rates in the stage wherein reaction force increases and in the stage wherein the reaction force is maintained constant.

**Figure 10:**
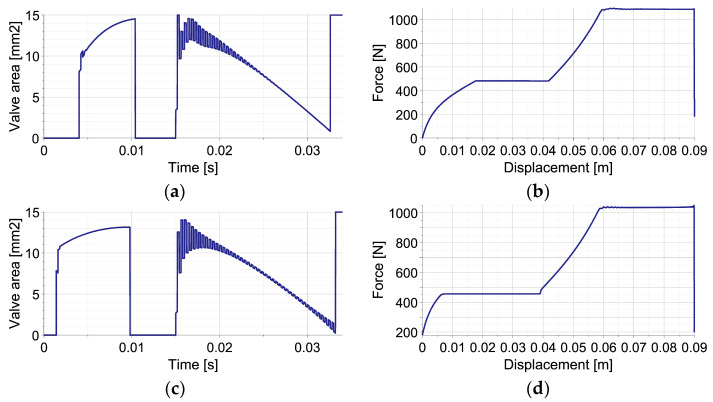
Mitigation of double-impact with disturbance using EPPC (non-perfectly inelastic collision): (**a**,**b**) valve opening area and generated reaction force in the case elastic disturbance; (**c**,**d**) valve opening area and generated reaction force in the case viscous disturbance.

In the second numerical example, the damping coefficient of the contact interface is assumed to be relatively small, which means that the impact is non-perfectly inelastic. The first considered case is the joint occurrence of partially inelastic collision and the elastic disturbance force acting along absorber’s stroke (Figure 10a,b). First, it can be observed that despite small contact damping, the rapid changes of valve opening required after the second impact gradually decrease (Figure 10a). Thus, the elastic disturbance force causes the positive effect of control process stabilization. Second, despite the presence of additional elastic force of unknown value, the total value of force generated by the absorber is successfully maintained as approximately constant both after the first and the second impact (Figure 10b).

The second considered case (Figure 10c,d) analyzes the joint occurrence of partially inelastic collision and viscous disturbance force. It can be observed that rapid changes of valve opening after the second impact gradually decrease initially, and rise at the end of the process (Figure 10a). Thus, the effect of control process stabilization occurs but declines due to the reduction of piston velocity and the corresponding viscous force along the absorber’s stroke. Moreover, the conducted simulations show that the joint presence of partially inelastic collision and the viscous disturbance force does not violate the operation of the proposed control method. After both impacts, the total generated force is effectively kept as approximately constant, and both objects are stopped at the end of the absorber stroke (Figure 10d).

**Figure 11:**
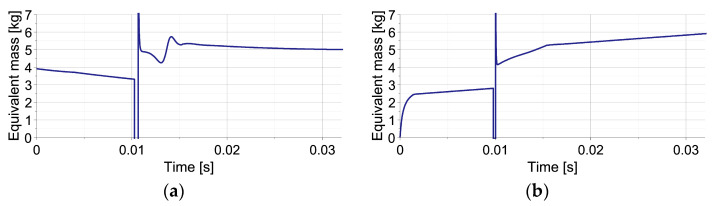
The change of equivalent mass during double-impact scenarios forces presented in Figure 10: (**a**) elastic disturbance force, (**b**) viscous disturbance force.

Both in the cases of elastic and viscous disturbances, during the first impact, the equivalent mass ($M_{\mathrm{eq}})$ changes analogously to the case of single-impact scenario (cf. Figure 4a,b and Figure 11a,b). However, in the case of elastic disturbance during the second impact, its value temporarily reaches infinite value, has some fluctuations due to sudden changes of the contact force, and further gradually decreases (Figure 11a). In turn, in the case of viscous disturbance during the second impact, the change of equivalent mass is more regular, but its increase occurs at different rates at both stages of the process (Figure 11b).

The above numerical simulations prove that EPPC with the sub-optimal analytical control strategy operates correctly when the double-impact scenario is disturbed by the occurrence of various types of additional forces. Despite the lack of knowledge of such disturbance forces, the method provides absorption of the entire kinetic energy of both impacting objects, utilization of the entire absorber’s stroke, and the minimal level of generated pneumatic force. On the other hand, the presented examples show that in the case of the low damping of the contact interface, the important feature of the proposed system design is the application of additional (e.g., elastic or viscous) elements inside the absorber. Although the mechanical characteristics of these elements do not have to be known, they result in simpler and more feasible in practice realization of the control process. A potential further challenge is designing of the additional element in such a way that the control process is realizable using the actuator of the assumed characteristics.

## 6. Conclusions

Derived and thoroughly analyzed in this paper, equivalent parameter predictive control has been proved to be an efficient method for the control of pneumatic absorbers subjected to various types of impact excitations under the influence of unknown disturbances. The successful operation of the method results from the combination of the adaptivity paradigm and the concept of model predictive control. The adaptivity is provided by the repetitive identification of the equivalent mass which substitutes both the unknown impacting object mass and the unknown system disturbances. In turn, the MPC approach with a repetitively updated predictive model and global energy absorption condition enables fast computation of optimal valve opening at each control step and efficient tracking of the optimal system path. The application of the proposed EPPC method provides the pneumatic absorber equipped with a controllable valve with the features of a self-adaptive system. It ensures automatic adaptation to the unknown mass of the impacting objects, unknown external forces, unknown disturbances, and double-impact excitations. In each case, the method enables the dissipation of the entire impact energy with the minimal level of generated force and the minimal level of the impacting object’s deceleration.

The successful implementation of the EPPC is based on the application of the sub-optimal analytical control strategy, which utilizes parameterized analytical functions describing the change of valve opening and the corresponding reaction force of the absorber. The arbitrary choice of these functions provides high accuracy of the control method, which is comparable as in the case of the optimal control strategy. In turn, their analytical form enables relatively simple and computationally efficient solutions of the constrained optimization problem. Finally, the continuity and smoothness of assumed functions allow us to avoid jumps of valve opening within a single control step and to stabilize the control actions required during the entire process.

The presented numerical verification of the EPPC operation clearly reveals that the method provides efficient impact mitigation in the case of a single-impact scenario, a double-impact scenario with various excitations, and a double-impact scenario with various disturbances. In particular, the numerical example concerning an unknown single impact (Section 5.1) proves that the proposed sub-optimal analytical control strategy ensures a similar response of the impact absorbing system as the optimal control strategy but uses a much smoother change of control signal and valve opening. Moreover, the numerical example involving an unknown double impact (Section 5.2) reveals that the proposed method works efficiently in the case of subsequent impacts of two objects with different time intervals, and its operation is not disrupted by the non-perfectly inelastic properties of the interface. Ultimately, the numerical example covering an unknown double impact with disturbances (Section 5.3) proves that the proposed control method remains robust in the case of unknown elastic and viscous disturbance forces occurrence, and, moreover, that intentionally introduced additional forces can provide stabilization of the control and system response.

The proposed equivalent parameter predictive control is expected to be applicable in many engineering problems, including landing gears, suspensions of cars, and mitigation of impacts during industrial processes. The practical realization of the proposed control method will be based on measurements of pneumatic force obtained via pressure sensors, measurements of impacting object deceleration via accelerometers, and control of the fluid flow via fast-operating (e.g., piezoelectric) valves. The proposed sensor-based approach to impact mitigation problems will introduce new challenges related to effective online measurement of the dynamic system state. Specifically, the impact process is relatively short and the measured quantities are unsteady and rapidly changing, so the measurements may be prone to significant errors. Therefore, high-frequency and accurate measurements of the system state will be essential for system operation. The specific requirements for the applied sensors and actuators strongly depend on the problem under consideration (especially impacting object velocity, impact period, and available stroke) and can be deduced from the developed mathematical model of the analyzed fluid-based damper and the proposed control system. Although such requirements seem to be very challenging, the recent intensive development of fast sensors and actuators based on smart materials, as well as fast control electronics utilizing FPGA platforms, indicates that the realization of such strategies will be possible in the very near future.

## Figures and Tables

**Table 1:** Comparison of control methods applied for fluid-based dampers and actuators.

	Unknown $\mathrm{Excitation}\;(M,\;v_{0}$)	Unknown Disturbance Force	Unknown Fluid Leakage	Actuator Time Delay	Actuator Fault	Comments
**Standard AIA** **approach [35]**	No	No	No	No	No	Precomputed system path
**Hybrid Prediction** **Control [42,43]**	Yes	Yes	Yes	No	No	Updated system path, approx. solution
**Identification-based** **Predictive Control [44,45]**	No/Yes *	Yes	No/Yes *	No/Yes *	No	Updated system path, semi-optimal solution
**Equivalent Parameter Predictive Control**	Yes	Yes	No	No	No	Updated system path, multi-impact loads, numerically efficient semi-optimal solution
**Robust Fault Tolerant Predictive Control [49]**	-	Yes	No	Yes	Yes	Application for industrial arm robot
**Adaptive Model Predictive Control [50]**	-	Yes	No	Yes	No	Application for system with uncertainties

* Depending on the version of the method.

**Table 2:** Parameters of the considered system.

Suspended Mass (kg)	Initial Velocity of the Mass (m/s)	Initial Internal Pressure in Chambers (kPa)	Operational Gas	Piston Diameter (mm)
5	5	300	compressed air	40
**Initial volume of top chamber (cm^3^)**	**Initial volume of bottom chamber (cm^3^)**	**Piston initial position (mm)**	**Entire absorber stroke (mm)**	**Initial** **temperature of the gas (K)**
7.54	118.12	6	94	293.15

**Table 3:** Basic parameters of the impact scenario.

First Impacting Mass (kg)	Initial Velocity of the First Mass (m/s)	Second Impacting Mass (kg)	Initial Velocity of the Second Mass (m/s)
4	4.5	2	5

## Data Availability

Upon reasonable request, data are available from the corresponding author pending approval by the Institute of Fundamental Technological Research, Polish Academy of Sciences.

## Abbreviations

The following abbreviations are used in this manuscript:

EPPC	Equivalent Parameter Predictive Control
AIA	Adaptive Impact Absorption
PID	Partial Integral–Differential
MPC	Model Predictive Control
HPC	Hybrid Prediction Control
IPC	Identification-based Predictive Control
FPGA	Field-programmable Gate Array

## Appendix A. Proof That Variational Formulation Given by Equation (33) Can Be Approximated by the Variational Formulation Given by Equation (34) under Specified Conditions

**Proof.** Let us assume that there exist exact solutions to the original and approximate variational problems given by Equations (33) and (34), defined by zero values of the integrand of each formulation. For the original path-tracking problem (Equation (33)), the solution reads as follows:

(A1) $$F_{p}\left(A_{v}\left(t\right)\right)+\left[\frac{F_{p}\left(t_{i}\right)}{{\ddot{u}}_{I}\left(t_{i}\right)}-\frac{F_{c}\left(t_{i}\right)+F_{\mathrm{ext}}\left(t_{i}\right)-F_{\mathrm{dist}}\left(t_{i}\right)}{{\ddot{u}}_{I}\left(t_{i}\right)}\right]\frac{{\dot{u}}_{I}{\left(t_{i}\right)}^{2}}{2\left(d-u_{I}\left(t_{i}\right)\right)}\;-\left[F_{c}\left(t_{i}\right)+F_{\mathrm{ext}}\left(t_{i}\right)-F_{\mathrm{dist}}\left(t_{i}\right)\right]=0,$$

While for the approximate path-tracking problem (Equation (34)), it takes the following form:

(A2) $$F_{p}\left(A_{v}\left(t\right)\right)+\frac{F_{p}\left(t_{i}\right)}{{\ddot{u}}_{I}\left(t_{i}\right)}\frac{{\dot{u}}_{I}{\left(t_{i}\right)}^{2}}{2\left(d-u_{I}\left(t_{i}\right)\right)}=0.$$

After simple rearrangement of terms in (A1), for the original formulation, one obtains the following:

(A3) $$-\frac{\frac{{\dot{u}}_{I}{\left(t_{i}\right)}^{2}}{2\left(d-u_{I}\left(t_{i}\right)\right)}}{{\ddot{u}}_{I}\left(t_{i}\right)}=\frac{\left[F_{c}\left(t_{i}\right)+F_{\mathrm{ext}}\left(t_{i}\right)-F_{\mathrm{dist}}\left(t_{i}\right)\right]-F_{p}\left(A_{v}\left(t\right)\right)}{\left[F_{c}\left(t_{i}\right)+F_{\mathrm{ext}}\left(t_{i}\right)-F_{\mathrm{dist}}\left(t_{i}\right)\right]-F_{p}\left(t_{i}\right)}$$

And, analogously, by transforming (A2), for the approximate formulation, one obtains the following:

(A4) $$-\frac{\frac{{\dot{u}}_{I}{\left(t_{i}\right)}^{2}}{2\left(d-u_{I}\left(t_{i}\right)\right)}}{{\ddot{u}}_{I}\left(t_{i}\right)}=\frac{F_{p}\left(A_{v}\left(t\right)\right)}{F_{p}\left(t_{i}\right)}.$$

In the considered problem, we are searching for the conditions under which the solution to the exact variational problem (A3) can be approximated by the solution to the approximate variational problem (A4). Thus, the problem is aimed at finding the conditions when the difference between the r.h.s. of (A3) and the r.h.s. of (A4) tends to zero:

(A5) $$\frac{\left[F_{c}\left(t_{i}\right)+F_{\mathrm{ext}}\left(t_{i}\right)-F_{\mathrm{dist}}\left(t_{i}\right)\right]-F_{p}\left(t\right)}{\left[F_{c}\left(t_{i}\right)+F_{\mathrm{ext}}\left(t_{i}\right)-F_{\mathrm{dist}}\left(t_{i}\right)\right]-F_{p}\left(t_{i}\right)}-\frac{F_{p}\left(t\right)}{F_{p}\left(t_{i}\right)}\to 0.$$

In order to conduct the proof, we assume the simplest possible formula defining the change of pneumatic force during a single control step in the following form:

(A6) $$F_{p}\left(t\right)=a\left(t\right)F_{p}\left(t_{i}\right),$$

where $a\left(t\right)$ is an arbitrary function of time. The exemplary relation of this type results from the adiabatic state of gas and takes the following form:

(A7) $$F_{p}\left(t\right)={\left[\frac{V\left(t_{i}\right)}{V\left(t\right)}\right]}^{\kappa }F_{p}\left(t_{i}\right).$$

Moreover, for the sake of simplicity, we define the quantity as follows:

(A8) $$F_{\mathrm{add}}\left(t_{i}\right)=F_{c}\left(t_{i}\right)+F_{\mathrm{ext}}\left(t_{i}\right)-F_{\mathrm{dist}}\left(t_{i}.\right)$$

By substituting Equations (A6) and (A8) into the left-hand side of the Equation (A5), we obtain the following:

(A9) $$\frac{F_{\mathrm{add}}\left(t_{i}\right)-F_{p}\left(t\right)}{F_{\mathrm{add}}\left(t_{i}\right)-F_{p}\left(t_{i}\right)}-\frac{F_{p}\left(t\right)}{F_{p}\left(t_{i}\right)}=\frac{F_{\mathrm{add}}\left(t_{i}\right)-{\mathrm{aF}}_{p}\left(t_{i}\right)}{F_{\mathrm{add}}\left(t_{i}\right)-F_{p}\left(t_{i}\right)}-a=\frac{F_{\mathrm{add}}\left(t_{i}\right)-{\mathrm{aF}}_{\mathrm{add}}\left(t_{i}\right)}{F_{\mathrm{add}}\left(t_{i}\right)-F_{p}\left(t_{i}\right)}=\frac{F_{\mathrm{add}}\left(t_{i}\right)\left(1-a\right)}{F_{\mathrm{add}}\left(t_{i}\right)-F_{p}\left(t_{i}\right)}$$

From Equation (A9), it can be clearly seen that the analyzed difference between the r.h.s. of Equation (A3) and the r.h.s. of Equation (A4) tends towards zero in two cases:

i. When the additional force in the system tends towards zero: $F_{\mathrm{add}}\left(t_{i}\right)\to 0$ (trivial case);
ii. When the coefficient defining the change of pneumatic force during a single control step tends towards one: a→1, which indicates a situation when the change of pneumatic force during the considered control step is relatively small.

This finishes the proof. □
